# CEST Contrasts Exhibit Significant Regional Variations in the Human Brain at 3 T

**DOI:** 10.1002/nbm.70177

**Published:** 2025-11-13

**Authors:** Florian Kroh, Philip S. Boyd, Svenja Graß, Nikolaus von Knebel Doeberitz, Heinz‐Peter Schlemmer, Mark E. Ladd, Daniel Paech, Andreas Korzowski

**Affiliations:** ^1^ Division of Medical Physics in Radiology German Cancer Research Center (DKFZ) Heidelberg Germany; ^2^ Department of Radiology Brigham and Woman's Hospital, Harvard Medical School Boston USA; ^3^ Faculty of Physics and Astronomy Heidelberg University Heidelberg Germany; ^4^ Division of Radiology German Cancer Research Center (DKFZ) Heidelberg Germany; ^5^ Faculty of Medicine Heidelberg University Heidelberg Germany

**Keywords:** APT, brain atlas, CEST, gray matter, rNOE, ssMT, white matter

## Abstract

Chemical exchange saturation transfer (CEST) MRI is a promising molecular imaging technique with established clinical relevance in neuro‐oncology. While CEST contrast differences between gray matter (GM) and white matter (WM) are documented, brain region–specific contrast variations remain underexplored. This study investigates the regional variability of CEST contrasts in healthy brains to provide a baseline reference, which could enhance the detection of subtle pathological changes in clinical settings. Ten healthy volunteers (five female, mean age 25 ± 3.1 years) underwent 3D CEST imaging on a 3‐T Siemens Prisma scanner. Using a custom segmentation tool, GM and WM regions of interest (ROIs) were automatically selected in the frontal, parietotemporal, and occipital regions and the calcarine sulcus to analyze regional contrast changes for the relaxation‐compensated MTR_Rex_ and asymmetry‐based APTw CEST contrasts. Individual and grouped analyses showed significant regional differences in GM and WM for all CEST contrasts. Globally, significant GM‐WM differences were also detected for the APTw, MTR_Rex_ AMIDE, and MTR_Rex_ ssMT, which demonstrated higher GM contrast values for APTw and MTR_Rex_ AMIDE and lower GM contrast values for the MTR_Rex_ ssMT. Regionally, all contrasts showed reduced GM signals in the frontal lobe and increased signals in the calcarine sulcus when compared to the occipital and parietotemporal lobe; however, these differences were less pronounced for MTR_Rex_ rNOE and MTR_Rex_ ssMT. Relaxation‐compensated CEST and APTw CEST contrast values exhibit significant regional variation in the healthy brain, highlighting the importance of consistent ROI placement in clinical studies. At the same time, low intersubject variability was observed, providing robust normative values for future comparisons. These regional reference values can aid in the detection of subtle pathological changes in CEST MRI by offering a reliable baseline for interpreting deviations in patient data.

## Graphical abstract

Both relaxation‐compensated and asymmetry‐based APTw CEST contrasts show significant regional variability in healthy human brains.

These findings expand our knowledge about CEST contrasts and highlight the need for consistent placement of control ROIs in neuroimaging, which is particularly crucial in pathologies that only lead to subtle differences in CEST contrast.

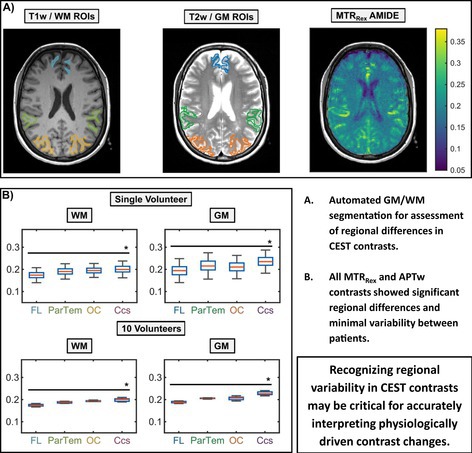

**Abbreviations**
APTw: amide proton transfer weighted
AREX: apparent exchange‐dependent relaxation
Ccs: calcarine sulcus
CEST: chemical exchange saturation transfer
CSF: cerebrospinal fluid
CV: coefficient of variation
DS: direct water saturation
FL: frontal lobe
FoV: field of view
GM: gray matter
GRAPPA: generalized autocalibrating partial parallel acquisition
LD: Lorentzian difference
MRI: magnetic resonance imaging
MITK: Medical Imaging Interaction Toolkit
MTR_Rex_
: inverse metric yielding R_ex_
OC: occipital lobe
ParTem: parietotemporal region
RF: radio frequency
rNOE: exchange‐relayed nuclear Overhauser effect
ROI: region of interest
SD: standard deviation
SPM: statistical parametric mapping
ssMT: semi‐solid magnetization transfer
WASABI: water shift and B_1_
WM: white matter

## 1 Introduction

Chemical exchange saturation transfer (CEST) magnetic resonance imaging (MRI) is an emerging molecular imaging technique that exploits the exchange of protons between specific endogenous biomolecules and water, resulting in contrasts reflecting the underlying molecular composition of tissues [1, 2, 3]. Unlike conventional MRI, which primarily provides anatomical images based on water proton density and relaxation times, CEST MRI is uniquely sensitive to the presence and concentration of various metabolites, proteins, and other biomolecules [3, 4]. This sensitivity makes CEST MRI particularly valuable for detecting and characterizing subtle biochemical changes that occur in various physiological and pathological states, especially within the brain.

In the neuro‐oncological context, CEST MRI has already demonstrated its clinical relevance by revealing altered molecular signatures in tissues affected by disease, such as tumors, where the contrast generated by CEST is significantly different from that of healthy brain tissue (gray matter [GM] and white matter [WM]) and associated with tumor subtypes [5, 6, 7] as well as response to therapy [8, 9, 10, 11, 12] and survival [13, 14]. Beyond oncology, other pathological conditions, including neurological, psychiatric, and inflammatory disorders, also induce molecular changes that can be effectively interrogated using CEST MRI [15, 16, 17]. However, in cases where pathological changes lead to only slight alterations in CEST effects—such as regions with a low volume fraction of tumor cells or early‐stage disease—these subtle differences might go undetected, potentially limiting the diagnostic significance of CEST MRI. Among the various CEST contrasts, amide proton transfer–weighted (APTw) imaging has gained particular attention due to its relatively short acquisition time and promising results in numerous clinical studies. From a clinical perspective, APTw is especially attractive because of its feasibility and reproducibility. However, APTw is not a pure amide signal—it is a weighted contrast influenced by multiple contributors, including amide protons, exchange‐relayed nuclear Overhauser effect (rNOE), and semi‐solid magnetization transfer (ssMT), as highlighted in recent consensus recommendations [18]. To better understand and interpret the composite nature of APTw contrast, it is essential to investigate its individual contributors. This is possible using more quantitative and acquisition‐intensive CEST approaches such as MTR_Rex_ and AREX based on the Lorentzian difference (LD) method, which allows for the relative isolation of specific Z‐spectrum components like rNOE, AMIDE, and ssMT. While these techniques offer greater specificity, they come with longer acquisition times and more complex post‐processing, making them currently less suited for routine clinical use. Still, they provide valuable insight into the biophysical mechanisms underlying CEST signal changes and thus are critical for validating and interpreting APTw findings.

The distinction between GM and WM in terms of CEST contrast is well‐documented, originating from the differing molecular compositions and properties of these tissues [19, 20, 21, 22]. It is therefore reasonable to hypothesize that different regions of the human brain, such as the frontal and occipital lobes, may also exhibit distinct CEST contrasts due to their specific functions and metabolic activities. Despite this plausible regional variability, there is currently no comprehensive description of how CEST contrast values vary across different brain regions in healthy individuals. This gap in knowledge poses a challenge for the interpretation of CEST MRI, particularly when trying to distinguish subtle pathological changes from normal regional variations.

Establishing a detailed understanding of brain region–specific differences in CEST contrasts is thus crucial for defining healthy reference values, which could significantly enhance the ability to detect and interpret subtle pathological changes. By providing a baseline of what constitutes normal CEST contrast values in various brain regions, clinicians and researchers would be better equipped to identify deviations that may indicate early or mild disease, thereby increasing the clinical utility of CEST MRI.

The purpose of this study was to investigate the regional variability of CEST contrasts in the healthy human brain. Specifically, we aimed to quantify the differences in CEST contrasts across various brain regions using 3‐T MRI in a cohort of 10 young, healthy volunteers. By segmenting the brain into distinct regions based on a standard brain atlas [23, 24], we analyzed different CEST contrasts for brain region–specific contrast alterations. Through the initial establishment of healthy reference values for regional varying CEST contrasts, this work seeks to improve the detection of nuanced pathological effects and expand the clinical relevance of CEST MRI.

## 2 Methods

### 2.1 Experimental Setup

Ten healthy and age‐matched volunteers (five female) with a mean age of 25 ± 3.1 years (male: 25 ± 1.9 years; female: 25 ± 4.1 years) were selected for this study. All examinations were approved by the local ethics committee of the Medical Faculty of Heidelberg University, and written informed consent was received from all subjects.

The CEST MRI measurements were performed using a 3‐T whole‐body MR scanner (Siemens; MAGNETOM Prisma) with an integrated transmit body coil, a 64‐channel receive head/neck coil, and a 3D spiral centric reordered gradient echo acquisition [25]. The readout and presaturation parameters were adapted from literature [26] and optimized for the used setup by Goerke et al. [20] in previous work. The applied readout settings were as follows: matrix = 128 × 104 × 16, resolution = 1.7 × 1.7 × 3 mm^3^, FOV = 21.76 × 17.68 × 4.80 cm^3^, GRAPPA acceleration factor = 2, TE = 2.75 ms, TR = 5.5 ms, bandwidth = 340 Hz/pixel, flip angle = 7°, and an elongation factor of 0.5, leading to an acquisition duration of t_acq_ = 3.6 s per readout.

Since CEST MRI signals are highly sensitive to the selected saturation parameters (pulse shape, saturation power B_1_, saturation time t_sat_, duty cycle, etc., as detailed below) that sometimes only certain evaluation methods are allowed, the protocol was optimized to incorporate multiple saturation schemes. Specifically, two low‐power (B_1_ of 0.6 and 0.9 μT), whole–Z‐spectrum CEST series were included (t_tot_ = 7:36 min each), and a conventional APTw CEST scan was performed (B_1_ = 2 μT; t_tot_ = 2:00 min). Furthermore, a WASABI scan [27] for mapping of B_1_ and B_0_ inhomogeneities (t_tot_ = 3:41 min) and a T_1_ saturation recovery scan [28] for quantitative T_1_ determination (t_tot_ = 1:58 min) were acquired, leading to a total acquisition time of 22:51 min for all CEST‐related measurements. All measurements were performed using the image acquisition parameters identical to those used for the CEST MRI data acquisition.

Presaturation for the two low‐power CEST series was realized using 148 Gaussian‐shaped RF pulses with a pulse duration of t_p_ = 20 ms, a mean amplitude of B_1_ = flip angle/(γ·t_p_) = 0.6 or 0.9 μT, and a duty cycle of DC = 80%, leading to a total saturation time of t_sat_ = 3.7 s. Furthermore, 57 frequency offsets were acquired, unequally distributed between ± 250 ppm for each CEST series (the complete list of acquired frequency offsets can be found in the SI of the original publication of the state‐of‐the‐art protocol [20]). For normalization, two M_0_'s (equilibrium water magnetization) were acquired per CEST series (Δω = −300 ppm), i.e., one at the beginning and one at the end.

The presaturation for the conventional APTw CEST measurement was achieved according to Zhou et al. [29] in line with the consensus guidelines [18] by applying a total of four rectangular RF pulses with a pulse duration of 200 ms, a B_1_ of 2 μT, and a duty cycle of 95% (t_sat_ = 830 ms). For normalization, one M_0_ was acquired at the beginning (Δω = −300 ppm) of the acquisition followed by 16 frequency offsets that were acquired focused around the −3.5 and +3.5 ppm offsets in the following manner: ± 4 ppm (one repetition [rep]), ± 3.75 ppm (two reps), ± 3.5 ppm (two reps), ± 3.25 ppm (two reps), and ± 3 ppm (one rep).

### 2.2 Data Processing

All CEST postprocessing was performed using in‐house developed scripts in MATLAB (2019b, The MathWorks Inc., Natick, MA, USA). At first, the APTw CEST, WASABI, and T_1_ images were co‐registered using an intensity‐based rigid image co‐registration algorithm in the Medical Imaging Interaction Toolkit (MITK) [30]. For the low‐power CEST data, a simple extension to the co‐registration algorithm proposed by Breitling et al. [31] was used enabling the identification and mitigation of direct water saturation (DS) artifacts. Following this, the WASABI measurement was evaluated as described by Schuenke et al. [27] to obtain B_0_ and rel. B_1_ maps, and the T_1_ inversion recovery data were evaluated to retrieve the quantitative T_1_ maps. In the next step, the CEST data were normalized using a voxel‐wise interpolation of the two acquired M_0_ to obtain an individual M_0_(Δω) for each frequency offset Δω, enabling the Z‐spectra normalization via Z(Δ) = M_sat_(Δ)/M_0_(Δ), where M_sat_(Δ) is the water magnetization after presaturation at a frequency offset Δω. After normalization, the CEST data (low power and APTw) were corrected for B_0_ inhomogeneities by shifting it along the frequency offset dimension according to the B_0_ map obtained from the WASABI measurement. For the APTw CEST data, the APTw contrast was calculated via

$$\text{APTw}=Z\left(-3.5\mathrm{ppm}\right)-Z\left(3.5\mathrm{ppm}\right).$$

This contrast was then used to calculate the fluid‐suppressed APTw contrast as described by Schüre et al. [32], which is intended to improve the readability of the APTw contrast maps by suppressing fluid contributions:

$$\text{APTw fluidsupp}=\text{APTw}*\frac{0.35^{2}}{Z{\left(-3.5\mathrm{ppm}\right)}^{2}}$$

For the calculation of the relaxation‐compensated MTR_Rex_ contrasts, four additional postprocessing steps were performed: (1) Z‐spectra were denoised, which was achieved through a principal component analysis‐based algorithm as described in Breitling et al. [33]. (2) Z‐spectra were fitted using a four‐pool Lorentzian fit model containing a pool for amides (Δ=3.5 ppm), DS (Δ=0 ppm), ssMT (Δ = −2.5 ppm), and rNOE (Δ=−3.5 ppm). Fitting was performed voxel‐wise and due to the pulsed presaturation a constant plateau was added to the DS resonance [26] (a detailed description of the fit model, the complete list of fit parameters containing starting values, upper and lower boundaries can be found in the Supporting Information of the original publication of the state‐of‐the‐art protocol [20] and representative Z‐spectra with fit are provided in Figure S1). Performing the Lorentzian fit enabled the calculation of a labeled Z‐spectrum (Z_lab_; i.e., all fitted pools) and a reference Z‐spectrum (Z_ref_; i.e., the pool of interest is excluded) for each pool. (3) The Z_lab_ and Z_ref_ were then used to calculate the relaxation‐compensated contrast MTR_Rex_ [34, 35] for the AMIDE (Δ = +3.5 ppm), rNOE (Δ = −3.5 ppm), and the ssMT (Δ = −2.5 ppm) pool as follows:

$$\mathrm{MT}R_{\mathrm{Rex}}\;\left(\Delta \omega \right)=1/Z_{\mathrm{lab}}\left(\Delta \omega \right)-1/Z_{\mathrm{ref}}\left(\Delta \omega \right).$$

To correct for B_1_ inhomogeneities, (4) the MTR_Rex_ contrasts were corrected voxel‐wise using the relative B_1_ map obtained from the WASABI measurement and the two‐point contrast B_1_ correction [36]. The B_1_‐corrected MTR_Rex_ contrasts were reconstructed at a B_1_ of 0.7 μT. Furthermore, the non–relaxation‐compensated LD and the relaxation‐compensated AREX contrasts [35] were calculated, the latter additionally allowing a compensation for the scaling of all CEST effects by the T_1_ relaxation time provided by quantitative T_1_ maps as follows:

$$\mathrm{LD}\left(\Delta \omega \right)=Z_{\mathrm{ref}}\left(\Delta \omega \right)-Z_{\mathrm{lab}}\left(\Delta \omega \right),$$

$$\text{AREX}\left(\Delta \omega \right)=\mathrm{MT}R_{\mathrm{Rex}}\;\left(\Delta \omega \right)/T_{1}.$$

After contrast calculation, the LD and AREX contrasts were B_1_‐corrected using the two‐point contrast B_1_ correction method in analogy to the MTR_Rex_ contrasts.

### 2.3 Brain Atlas–Based ROI Analysis

**FIGURE 1:**
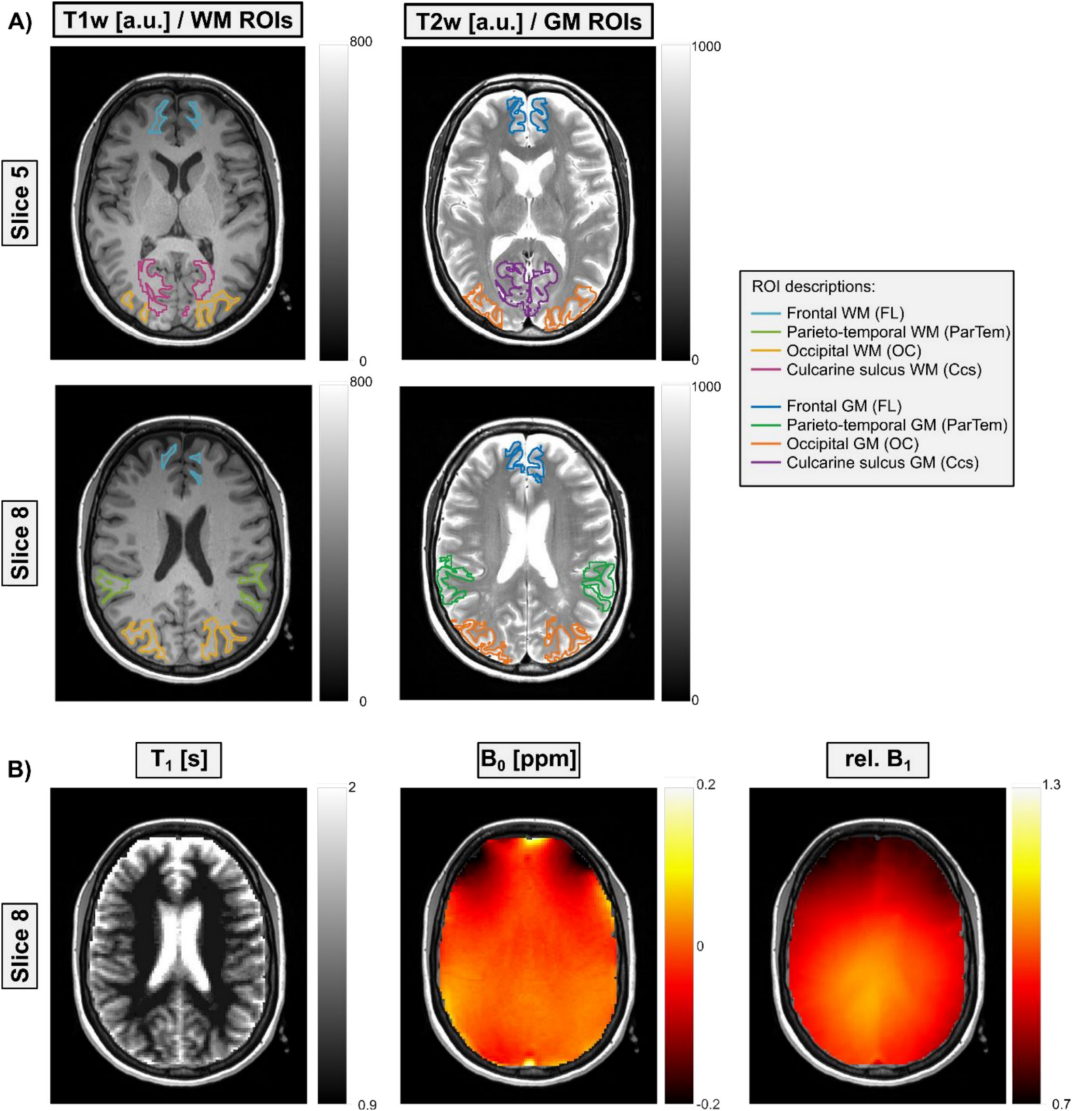
Illustration of representative gray matter (GM) and white matter (WM) masks as well as a T_1_, B_0_, and rel. B_1_ maps. (A) The GM ROIs for a representative volunteer are shown as overlays on a T_1_‐weighted image for two slices (Slice 5 top and Slice 8 bottom, total of 12 slices). The same is shown for the WM ROIs, which are overlaid on the T_2_‐weighted image for the same slices and volunteer. For GM and WM, the ROIs were created for the frontal lobe (FL), parietotemporal region (ParTem), occipital lobe (OC), and calcarine sulcus (Ccs) each (different colors). (B) The corresponding quantitative T_1_ map and B_0_ and rel. B_1_ maps of Slice 8 are displayed to give an impression of the data quality and possible confounding influences due to field inhomogeneities.

To enable the automated 3D segmentation of the volunteers' brains, T_1w_ MP‐RAGE (matrix = 640 × 640 × 176, voxel size = 0.4 × 0.4 × 1.0 mm^3^) and T_2w_‐TSE (matrix = 896 × 672 × 45, voxel size = 0.3 × 0.3 × 3.0 mm^3^) images were acquired for all volunteers. Based on these images, GM and WM ROIs were automatically selected using the unified segmentation framework, which is incorporated into SPM [23]. The probabilistic framework is based on the combination of a tissue classification approach and a registration via template approach. After segmentation, the GM and WM ROIs were combined with the brain atlas information from the SPM extension Automatic Anatomical Labeling Atlas 3 [24] to generate a GM and WM ROI in the frontal lobe (FL), parietotemporal region (ParTem), occipital lobe (OC), and calcarine sulcus (Ccs). Finally, the ROIs were mapped on the CEST contrast map geometry to enable the ROI analysis. Representative atlas‐based ROIs are illustrated in Figure 1. The ROI selection was guided by both technical and biological considerations. Technically, only regions containing at least 100 voxels per volunteer were included, which limited the number of possible ROIs due to the restricted field of view. Biologically, the goal was to ensure broad anatomical coverage by including at least one ROI from each major brain lobe, leading to the inclusion of FL, ParTem, OC, and Ccs.

### 2.4 Statistical Analyses

The Shapiro–Wilk test indicated that the contrast values within the regions were not normally distributed for individual participants and for the pooled median values. Consequently, Mann–Whitney *U*‐tests were performed to assess differences in CEST contrast values, i.e., both within individual participants and for the pooled median values. Each ROI was compared with every other ROI within its respective tissue type, and the overall GM and WM ROIs were likewise contrasted. Finally, coefficients of variation (CVs) were calculated for T_1_, LD, MTR_Rex_, and AREX contrasts for all ROIs in the pooled median value analysis to assess the variability relative to the mean.

All statistical analyses were conducted using custom‐built in‐house software in MATLAB (MathWorks, version 2019b).

## 3 Results

**FIGURE 2:**
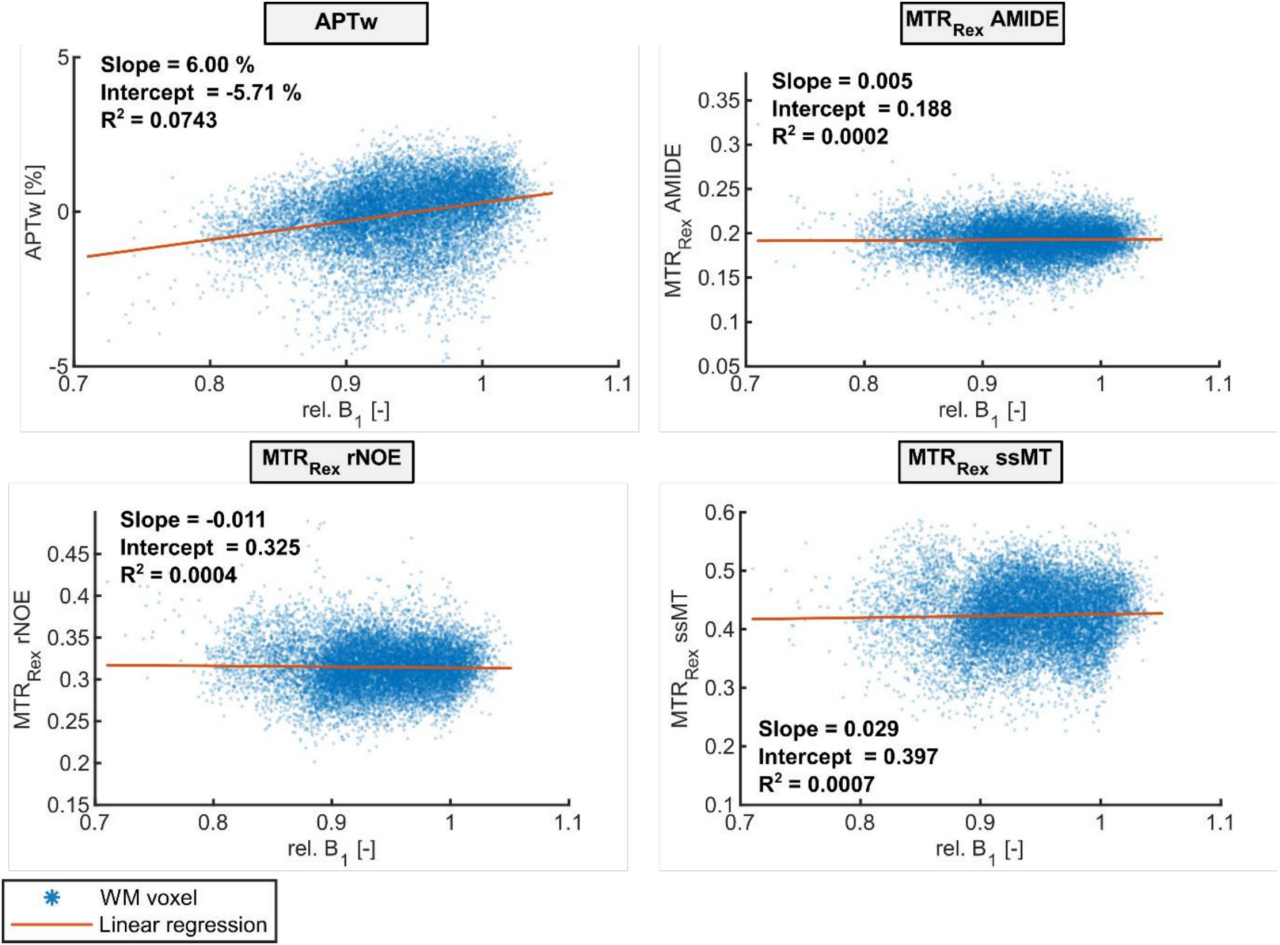
Voxel‐wise correlation analysis of the APTw, MTR_Rex_ AMIDE, MTR_Rex_ rNOE, and MTR_Rex_ ssMT contrast values with the relative B_1_ map. In the case of no correlation, the slope of the linear regression (orange line) and the coefficient of determination (*R* ^2^) should be zero. The correlation analysis was conducted for WM occipital lobe voxels of all volunteers. The analysis was performed only in this one region as one would suspect the same contrast values throughout the entire ROI and furthermore the selected ROI depicted the highest variation of rel. B_1_ values. Looking at the scatter plots and slopes as well as coefficients of determination, no correlation can be observed for the B_1_‐corrected MTR_Rex_ contrasts. However, a small tendency towards higher values for higher rel. B_1_ values could be observed for the APTw contrast.

To enable regional analyses of CEST contrasts, masks for GM and WM were created and combined with the SPM extension's Automatic Anatomical Labeling Atlas 3 [24], as outlined in Section 2. This integration provides a fully labeled brain atlas, allowing us to generate GM and WM ROIs in the FL, ParTem, OC, and Ccs, as illustrated in Figure 1 for a representative volunteer. Figure 1 displays WM ROIs (left panel) on a T_1_‐weighted (T_1w_) image and GM ROIs (middle panel) on a T_2_‐weighted (T_2w_) image across two different slices. Additionally, a quantitative T_1_, B_0_, and rel. B_1_ map is provided to assess potential confounding effects from variations like field inhomogeneities. The T_1_ map shows the expected pattern, with longer T_1_ relaxation times in GM regions. B_0_ inhomogeneities within evaluated volunteers were minimal, showing a mean shift of ≤ 0.05 ppm across regions with a standard deviation (SD) below 0.05 ppm. B_1_ inhomogeneities exhibited larger regional variation, with the most prominent decrease in the FL WM ROI (mean relative B_1_ = 0.74 ± 0.03). However, all MTR_Rex_ contrasts were B_1_‐corrected in postprocessing, and correlation analyses between MTR_Rex_ contrast values and B_1_ in the occipital WM ROI show no coefficients of determination larger than *R* ^2^ = 0.0008 for any of the MTR_Rex_ contrasts (Figure 2). The coefficient of determination of the APTw contrast with rel. B_1_ on the other hand is increased but still only low with *R* ^2^ = 0.0743 in the same region. The correlation analysis was conducted for WM OC voxels of all volunteers as one would suspect the same contrast values throughout the entire ROI and the selected ROI depicted the highest variation of rel. B_1_ values throughout the evaluated regions.

**FIGURE 3:**
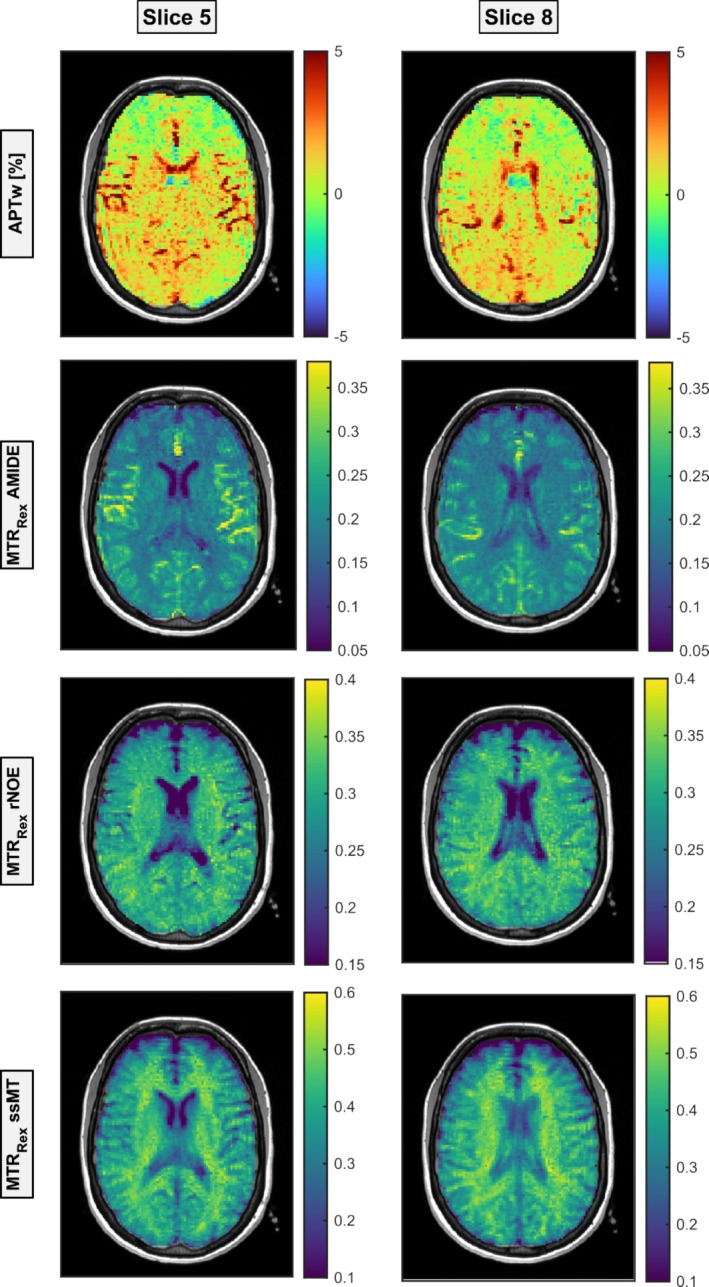
The same two slices as shown in Figure 1A are presented here to illustrate the visual characteristics of the analyzed contrasts: APTw (first row), relaxation‐compensated MTR_Rex_ AMIDE (second row), rNOE (third row), and ssMT (fourth row). APTw and MTR_Rex_ AMIDE exhibit differences between gray matter (GM) and white matter (WM), with enhanced contrast in GM. In contrast, rNOE and ssMT demonstrate an opposite trend, with smallest differences observed for rNOE. Notably, the APTw contrast maps depict ringing artifacts in the parieto‐occipital regions.

A first visual analysis of the CEST contrasts (Figure 3) in regard to regional and GM–WM differences shows that the APTw contrast exhibited hyperintensities in GM compared to WM. Regional differences are not visually discernible; instead, APTw shows heightened signal variability in the FL and hyperintensities near cerebrospinal fluid (CSF) regions, likely from partial volume effects, leading to observed intensity variations. However, this contrast also shows prominent hyperintensities in areas consistent with large blood vessels and perivascular regions that appear to dominate the APTw signal in several regions. The MTR_Rex_ AMIDE contrast displays similar hyperintense signal in GM, with intensity decreasing toward the FL in GM and WM, and hyperintense vascular regions. Finally, both MTR_Rex_ rNOE and MTR_Rex_ ssMT contrasts show a hyperintensity in WM compared to GM; however, it is less clear for rNOE, and regional differences are not visible. Furthermore, the blood‐rich regions are not similarly highlighted in these contrasts.

**FIGURE 4:**
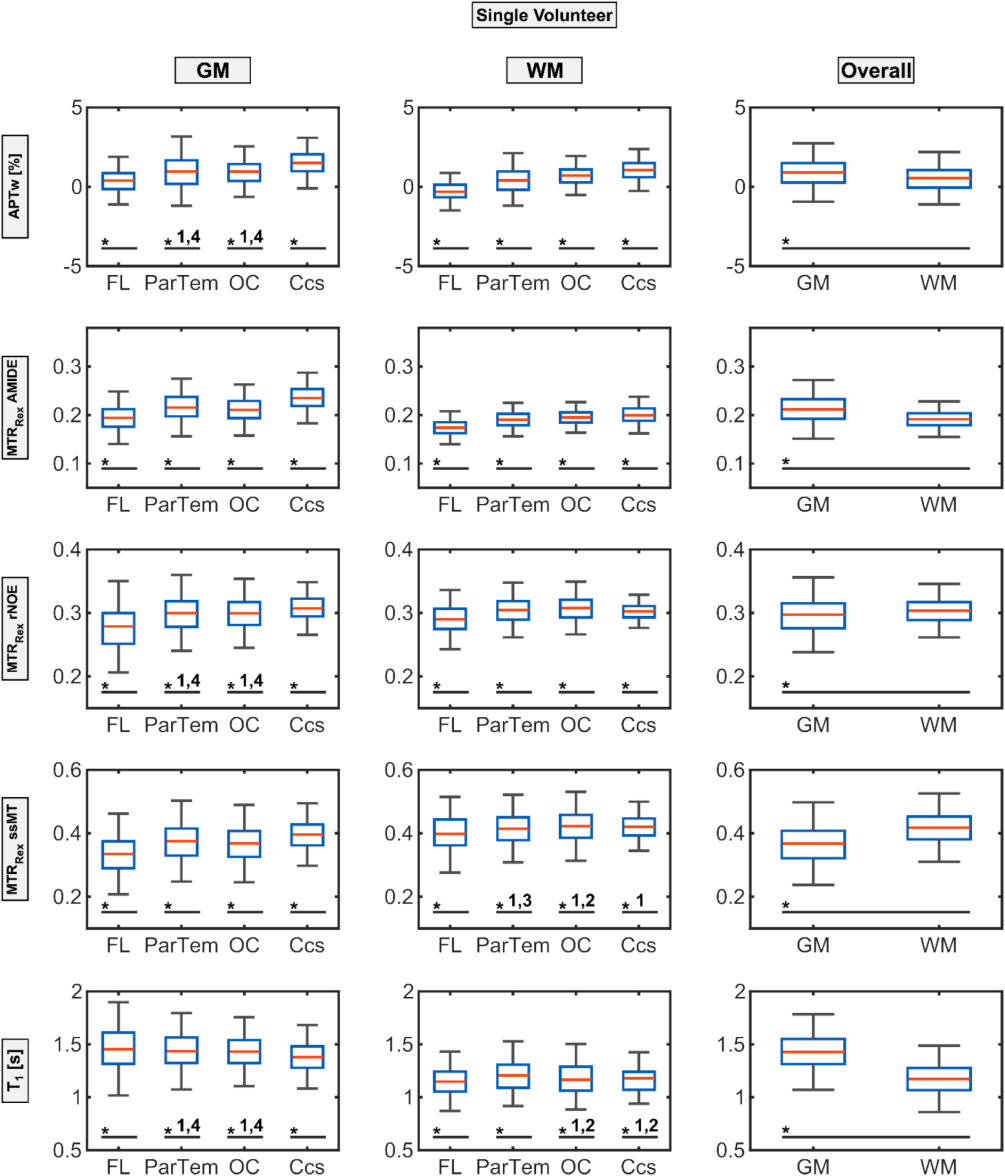
Boxplot showing the ROI analysis of the representative volunteer displayed in Figures 1 and 2. The APTw (first row) and relaxation‐compensated CEST contrast values (MTR_Rex_, Rows 2–4) as well as T_1_ times (fifth row) are displayed for the GM (left column) and WM (middle column) ROIs located in the frontal lobe (FL, 1) parietotemporal (ParTem, 2), occipital lobe (OC, 3), and the calcarine sulcus (Ccs, 4). Additionally, combined gray matter (GM) ROIs and combined white matter (WM) ROIs (right column) are displayed in the right column. A single asterisk (*) indicates that the marked group differs significantly (*p* < 0.05) from all other groups of ROIs within the subfigure. Where an asterisk is followed by a group number (e.g., 2), the marked group differs significantly (*p* < 0.05) only from that specific group. Note that a second representative volunteer is shown in the supporting information (Figure S2).

To further substantiate these observations, boxplot analyses with statistical significance tests as described in Section 2 were conducted for each ROI to evaluate potential regional GM and WM contrast value differences (Figure 4, Table 1). For the representative volunteer shown, APTw (GM: 0.90 ± 1.01, WM: 0.45 ± 0.82, *p* < 0.01) and MTR_Rex_ rNOE (GM: 0.29 ± 0.03, WM: 0.30 ± 0.02, *p* < 0.01) exhibited only minimal, yet statistically significant, GM–WM differences.

**TABLE 1:** Summary of the ROI analyses for the single volunteer displayed in Figure 4. Mean and standard deviation (SD) were calculated for APTw, MTR_Rex_ AMIDE, MTR_Rex_ rNOE, MTR_Rex_ ssMT, and the quantitative T_1_ measurement in the combined GM and WM ROIs as well as for the individual regions (FL = frontal lobe, ParTem = parietotemporal region, OC = occipital lobe, Ccs = calcarine sulcus).

	FL		ParTem		OC		Ccs		Combined	
	Mean	SD	Mean	SD	Mean	SD	Mean	SD	Mean	SD
GM										
APTw (%)	0.39	0.80	1.03	1.16	0.85	0.88	1.57	0.91	0.90	1.01
MTR_Rex_ AMIDE	0.20	0.03	0.22	0.03	0.21	0.03	0.24	0.03	0.21	0.03
MTR_Rex_ rNOE	0.27	0.04	0.30	0.03	0.30	0.03	0.31	0.02	0.29	0.03
MTR_Rex_ ssMT	0.33	0.06	0.37	0.06	0.36	0.06	0.39	0.05	0.36	0.06
T_1_ (s)	1.48	0.22	1.44	0.18	1.43	0.17	1.38	0.15	1.44	0.18
WM										
APTw (%)	−0.27	0.62	0.40	0.85	0.65	0.69	1.06	0.65	0.45	0.82
MTR_Rex_ AMIDE	0.17	0.02	0.19	0.02	0.20	0.02	0.20	0.02	0.19	0.02
MTR_Rex_ rNOE	0.29	0.02	0.30	0.02	0.31	0.02	0.30	0.01	0.30	0.02
MTR_Rex_ ssMT	0.40	0.06	0.41	0.05	0.42	0.05	0.42	0.04	0.42	0.05
T_1_ (s)	1.15	0.14	1.20	0.14	1.18	0.15	1.17	0.12	1.18	0.14

In contrast, MTR_Rex_ AMIDE and ssMT, as well as T_1_, showed strong and significant GM–WM differentiation: GM signal was higher for AMIDE (GM: 0.21 ± 0.03, WM: 0.19 ± 0.02, *p* < 0.01) and T_1_ (GM: 1.44 ± 0.18 s, WM: 1.18 ± 0.14 s, *p* < 0.01), whereas ssMT demonstrated a lower GM signal (GM: 0.36 ± 0.06 s, WM: 0.42 ± 0.05 s, *p* < 0.01).

Regionally, the quantitative T_1_ map displayed relatively modest yet, in most cases, still statistically significant variation, with GM mean values ranging from 1.38 s (Ccs) to 1.48 s (FL) and WM values from 1.15 s (FL) to 1.20 s (ParTem). However, the CEST contrasts showed more pronounced regional dependencies. Both APTw asymmetry and MTR_Rex_ AMIDE demonstrated distinct GM signal reductions in the FL and elevated signals in the Ccs when compared with the OC and ParTem. For APTw, the difference in mean values between the FL (GM: 0.39 ± 0.80, WM: −0.27 ± 0.62) and Ccs (GM: 1.57 ± 0.91, WM: 1.06 ± 0.65) exceeded 1 SD in GM and 2 SD in WM, and these values differed significantly (*p* < 0.01) from all other evaluated regions.

Similarly, MTR_Rex_ AMIDE showed a clear, significant regional variation in WM (GM FL: 0.20 ± 0.03, WM FL: 0.17 ± 0.02, GM Ccs: 0.24 ± 0.03, WM Ccs: 0.20 ± 0.02). In both GM and WM, MTR_Rex_ rNOE and ssMT also exhibited significant regional trends similar to APTw and MTR_Rex_ AMIDE, though they were less pronounced. Specifically, in GM, a similar increased signal in the Ccs and decreased signal in the FL relative to other regions and, in WM, the same direction of change but of smaller magnitude can be observed.

**FIGURE 5:**
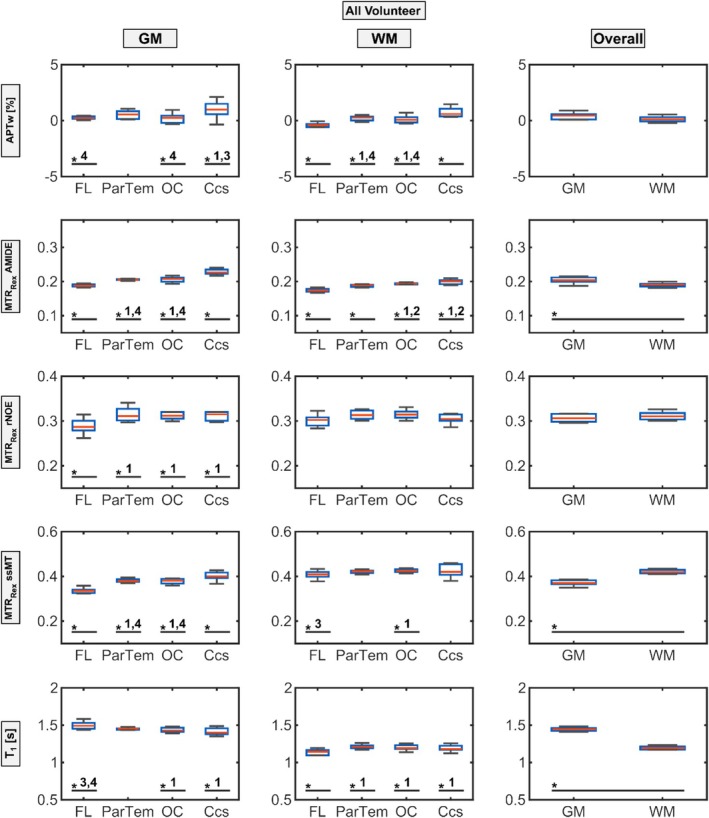
Boxplot of the median signal values for the APTw (first row) and relaxation‐compensated MTR_Rex_ contrasts (Rows 2–4) as well as T_1_ times (fifth row) of the 10 volunteers (25 ± 3.1 years, five female). Data are displayed for the GM (left column) and WM (middle column). ROIs located in the frontal lobe (FL, 1) parietotemporal region (ParTem, 2), occipital lobe (OC, 3), and the calcarine sulcus (Ccs, 4). The combined gray matter (GM) ROIs and combined white matter (WM) ROIs (right column) are displayed in the right column. A single asterisk (*) indicates that the marked group differs significantly (*p* < 0.05) from all other groups of ROIs within the subfigure. Where an asterisk is followed by a group number (e.g., 2), the marked group differs significantly (*p* < 0.05) only from that specific group.

These findings were supported in a second volunteer (Figure S2), but more importantly, similar trends were observed in the median ROI value analysis across all 10 volunteers (Figure 5 and Table 2). Again, significantly higher GM compared to WM contrast values were observed for MTR_Rex_ AMIDE (GM: 0.20 ± 0.01, WM: 0.19 ± 0.01, *p* < 0.01) and T_1_ (GM: 1.45 ± 0.04, WM: 1.19 ± 0.03, *p* < 0.01), whereas ssMT showed significantly lower values in GM than WM (GM: 0.37 ± 0.01, WM: 0.42 ± 0.01, *p* < 0.01). By contrast, APTw asymmetry (GM: 0.33 ± 0.50, WM: 0.03 ± 0.45) and MTR_Rex_ rNOE (GM: 0.31 ± 0.01, WM: 0.31 ± 0.01) exhibited slight, nonsignificant GM–WM differences.

**TABLE 2:** Summary of the median ROI value analysis across all 10 volunteers displayed in Figure 5. Mean and standard deviation (SD) were calculated for APTw, MTR_Rex_ AMIDE, MTR_Rex_ rNOE, MTR_Rex_ ssMT, and the quantitative T_1_ measurement in the combined GM and WM ROIs, as well as for the individual regions (FL = frontal lobe, ParTem = parietotemporal region, OC = occipital lobe, Ccs = calcarine sulcus). The statistics demonstrate high consistency between volunteers, with lower standard deviations compared to those from individual measurements (cf. Table 1).

	FL		ParTem		OC		Ccs		Combined	
	Mean	SD	Mean	SD	Mean	SD	Mean	SD	Mean	SD
GM										
APTw (%)	0.16	0.34	0.44	0.53	0.14	0.59	0.98	0.68	0.33	0.50
MTR_Rex_ AMIDE	0.19	0.01	0.205	0.01	0.21	0.01	0.23	0.01	0.20	0.01
MTR_Rex_ rNOE	0.29	0.02	0.31	0.01	0.31	0.01	0.31	0.02	0.31	0.01
MTR_Rex_ ssMT	0.34	0.01	0.38	0.01	0.38	0.01	0.40	0.02	0.37	0.01
T_1_ (s)	1.49	0.05	1.46	0.03	1.44	0.04	1.42	0.06	1.45	0.04
WM										
APTw (%)	−0.51	0.35	0.13	0.41	0.01	0.56	0.64	0.57	0.03	0.45
MTR_Rex_ AMIDE	0.17	0.01	0.19	0.01	0.19	0.01	0.20	0.01	0.19	0.01
MTR_Rex_ rNOE	0.30	0.02	0.31	0.01	0.31	0.01	0.31	0.01	0.31	0.01
MTR_Rex_ ssMT	0.41	0.02	0.42	0.01	0.43	0.01	0.42	0.03	0.42	0.01
T1 (s)	1.14	0.06	1.21	0.03	1.20	0.04	1.20	0.05	1.19	0.03

Furthermore, most regional differences also emerged in the combined analysis across volunteers (Figure 5). For MTR_Rex_ AMIDE (GM FL: 0.19 ± 0.01, WM FL: 0.17 ± 0.01, GM Ccs: 0.23 ± 0.01, WM Ccs: 0.20 ± 0.01) and APTw (GM FL: 0.16 ± 0.34, WM FL: −0.51 ± 0.35, GM Ccs: 0.98 ± 0.68, WM Ccs: 0.64 ± 0.57), prominent and significant FL signal decreases and Ccs increases were observed in WM and GM for MTR_Rex_ AMIDE and in WM only for APTw. MTR_Rex_ ssMT also matched the single‐volunteer findings by showing a significant increase in the Ccs and a decrease in the FL in GM (GM FL: 0.34 ± 0.01, GM Ccs: 0.40 ± 0.02); however, regional WM differences in ssMT were mostly non‐significant (WM FL: 0.41 ± 0.02, WM Ccs: 0.42 ± 0.03). MTR_Rex_ rNOE, on the other hand, no longer showed significantly elevated GM signals in the Ccs; it exhibited only a significant decrease in FL GM signals, with no significant differences among WM regions.

Additionally, the median contrast values across volunteers demonstrated high consistency and relatively low SDs and CV (Table S4) compared with individual measurements, suggesting minimal inter‐volunteer variability for CEST contrasts among age‐matched participants. This also suggests only small sex‐related differences, as confirmed in an additional analysis (Tables S1 and S2 and Figures S3 and S4). Finally, analysis of the fluid‐suppressed APTw contrast, LD AMIDE, rNOE, and ssMT and AREX AMIDE, rNOE, and ssMT (Figures S5, S6, and S7 and Table S3) revealed similar patterns, with the sole exception of a hyperintense overall WM signal relative to GM for the AREX AMIDE contrast; notably, the regional differences persisted for LD and AREX AMIDE; however, for the MTR_Rex_ rNOE, the reduced FL GM contrast compared to the other GM ROIs vanished for the LD rNOE but stayed consistent for the AREX rNOE.

## 4 Discussion

In this study, the regional variability of relaxation‐compensated and asymmetry‐based CEST contrasts within the human brain was systematically evaluated across an age‐matched cohort of 10 healthy volunteers (mean age 25 ± 3.1), including five males and five females. The goal was to assess whether subtle biological and anatomical differences in different brain regions are observable using CEST contrasts to potentially improve the detection of nuanced pathological effects. Below, the key findings and implications are discussed.

### 4.1 Robustness of CEST Measurements

This study demonstrated low variability in median CEST contrast values among the volunteers, indicating that CEST imaging is a reproducible tool for capturing contrast across brain regions in age‐matched participants. Similarly, a recent study in healthy volunteers by Cronin et al. [22] showed very good scan–rescan reproducibility for MTR_Rex_ rNOE and Lee et al. [37] as well as Pflueger et al. [38] showed very good reproducibility for the APTw contrast. This reliability is crucial for clinical and research applications using quantitative analysis, where consistent imaging is required to detect subtle changes or variations in brain tissue, especially in longitudinal studies. However, although the deviations in the median values among different subjects are very small, the contrast deviations in individual volunteers are increased, which limits the ability of the CEST contrasts to identify small pathological changes without ROI mean value analysis. Furthermore, there is a need to evaluate inter‐scanner and inter‐vendor variability, which should not be underestimated as shown by Voelker et al. [39].

Furthermore, no apparent sex‐related differences were observed across all contrasts in this cohort (see Tables S1 and S2 and Figures S3 and S4). While this suggests a degree of consistency in CEST imaging across sexes, the small sample size (*n* = 5 per sex) and narrow age range limit the generalizability of this finding. In particular, known physiological differences between sexes, such as hemoglobin concentration [40], warrant further investigation. Additionally, age can impact CEST contrast, as shown by Meinecke et al. [41], highlighting a potential confounder that could benefit from further investigation in different age groups to determine if the same consistency holds true across larger and more diverse populations.

### 4.2 Regional Variability of CEST Contrast

First, as proposed by different groups [19, 20, 21, 22], there is a clear and significant difference between GM and WM signal for most all evaluated CEST contrasts. The APTw and MTR_Rex_ AMIDE contrast show an increased GM signal compared to WM [22, 26] and the opposite is true for rNOE [42] and ssMT as can be seen in Figures 4 and 5. This is expected, as GM is suspected to have a higher content of mobile proteins; therefore, increasing the amide contrasts [43, 44] and WM has a higher lipid [45] and macromolecule concentration, leading to increased rNOE and ssMT contrasts. Building on that, however, there are also significant regional differences in CEST contrast values, indicating high sensitivity of specific CEST contrasts, such as MTR_Rex_ AMIDE and APTw asymmetry, to anatomical regions.

The distinct contrast patterns observed in the FL, ParTem, OC, and Ccs—decreased contrast values in the FL and increased contrast in the Ccs compared to the ParTem and OC—could originate from a multitude of different factors. Firstly, a difference in proton density could be one contributor since a reduced neuron density is suggested in the FL [46]. However, in the case of the APTw contrast, this change of proton density is not limited to amide protons, since the contrast is a weighted contrast that also includes contributions from rNOE and ssMT effects, which may add to the regional contrast variations [18]. Secondly, a difference in exchange rate based on regional pH changes would influence the contrast values. Platek et al. [47] explored this using ^31^P MRSI, observing a lower pH in the FL compared to the ParTem and OC, which could account for the reduced CEST contrasts. However, it should be noted that Platek et al. did not perform a pH quantification in the Ccs. Lastly, changes in the labeling efficiency could also lead to CEST contrast differences. To this end, an analysis focusing on the correlation between the analyzed contrasts and B_1_ was performed to ensure that changes in B_1_ and therefore labeling efficiency are not the reason for the local variations. The analysis demonstrated that B_1_ inhomogeneities did not influence the regional variations in B_1_‐corrected MTR_Rex_ contrasts. This conclusion is supported by the lack of a strong correlation between rel. B_1_ and the MTR_Rex_ contrasts within a single region (OC lobe GM), validating that the observed contrast changes are due to tissue‐specific differences, rather than B_1_‐related effects. This finding is particularly valuable in regions prone to B_1_ field variations and supports the use of the B_1_‐corrected MTR_Rex_ contrasts even in studies where B_1_ inhomogeneities are to be expected. In contrast, the APTw signal demonstrated a slight correlation with B_1_ field strength, warranting further investigation. This correlation suggests that the composition of the APTw contrast may change with varying B_1_ levels. Since the amide and rNOE proton fractions and their exchange rates should remain stable within a single brain region and tissue type, this observation points to changes in labeling efficiency as the probable cause of the B_1_ dependency.

The regional variations underscore the importance of accounting for anatomical context when normalizing and standardizing CEST data, as well as when choosing normal‐appearing brain tissue control regions—particularly in patients with infiltrative gliomas. Their relevance becomes especially clear when compared to a recent analysis by von Knebel Doeberitz et al. [14], which used CEST imaging to predict overall survival in 49 glioma patients at their first follow‐up after radiotherapy. In that study, the cohort was stratified into two groups based on mean contrast values above or below the group medians, followed by a Kaplan–Meier analysis to assess associations with overall survival. A strong link between APTw contrast and overall survival was observed. Notably, the median contrasts in the high‐ and low‐value groups for both APTw and all MTR_Rex_ contrasts were comparable in magnitude to those observed between FL and Ccs in GM. This finding underscores the importance of consistent control ROI placement and emphasizes the need for further exploration of the relationship between tumor location and contrast values.

### 4.3 Impact of T_1_ Differences and Fluid Compartments on CEST Contrast Variations

To furthermore validate that possibly varying T_1_ times are not causing the different contrast values in the brain regions, the relaxation‐compensated AREX contrasts were calculated. These contrasts exhibited patterns consistent with the MTR_Rex_ CEST contrasts, confirming that the regional variations are indeed not T_1_ related. However, this does not mean that T_1_ does not influence the contrasts since there is a clear observable difference between the corresponding MTR_Rex_ and AREX contrasts in GM and WM. Furthermore, it is noteworthy that the non–spillover‐corrected LD contrasts exhibited similar regional differences within GM and WM, as well as consistent global differences between GM and WM. This suggests that the observed regional variations are not primarily driven by spillover effects of the underlying contributions from ssMT and direct water saturation effects. Lastly, the recently emerging fluid‐suppressed APTw contrast [32, 48, 49] was also analyzed to identify possible confounding different ssMT contributions to the APTw contrast. This analysis showed, again, no differences, therefore excluding ssMT changes as a possible reason for the APTw contrast differences in the healthy human brain.

### 4.4 Potential Limitations and Areas for Further Research

While the findings are robust, several limitations were noted. First, partial volume effects and voxel mismatches may affect the precision of CEST contrast values, especially in regions adjacent to CSF where such effects can skew contrast values (e.g., seen by increased SD in GM compared to WM). Future research could aim to refine imaging protocols and explore additional correction methods for motion or field inhomogeneities to improve accuracy in these areas. Second, the potential contamination of the amide‐related contrasts by vascular signals must be considered. The APTw contrast is strongly influenced by blood content as previously reported [50, 51]. This is visible in the hyperintense regions shown in Figure 3, which correspond to areas of high vascularization. The high protein content of blood (e.g., albumin, hemoglobin) contributes to both the AMIDE and rNOE signals. Interestingly, while the AMIDE contrast (including MTR_Rex_, LD, and AREX) reflects this well, the rNOE contrast does not show the same degree of vascular signal, which may suggest contamination by ssMT. Future studies could focus on investigating methods to better separate vascular contributions from tissue‐derived CEST signals, especially in areas close to large vessels.

Third, the current study covered only a limited portion of the brain (4.8‐cm slab) and included four representative ROIs. While these regions provide meaningful insights, they do not fully capture the heterogeneity of CEST contrasts across the entire brain. Future work should aim to expand the field of view to enable the incorporation of a broader set of brain regions to better understand spatial variations, especially in clinically relevant areas.

Fourth, there are important considerations regarding the clinical feasibility of different CEST imaging approaches. APTw imaging benefits from short acquisition times (~2 min, excluding B_0_ mapping) and straightforward postprocessing, making it suitable for clinical workflows. However, the resulting contrast is complex to interpret, particularly in non‐characterized pathologies, and is prone to confounding effects such as blood contamination. In contrast, LD‐based methods (including MTR_Rex_ and AREX) offer improved interpretability by providing more specific quantification of underlying exchange mechanisms. Yet they require significantly longer acquisition times (e.g., 2 × 7:36 min for full Z‐spectra), additional B_0_ and B_1_ corrections, and in the case of AREX, a quantitative T_1_ map. Furthermore, the postprocessing remains complex and not currently feasible without expert‐level knowledge, limiting routine clinical application. This trade‐off between clinical practicality and biophysical specificity should guide future protocol development and standardization efforts.

Additionally, studies across larger and more diverse populations and assessments of contrast behavior in tumor regions throughout the brain could offer deeper insights. Such studies could enhance understanding of CEST contrast variability in various pathological contexts and ultimately promote an accurate interpretation and use of CEST contrast metrics. Lastly, analyzing potential contrast deviations in gliomas across different brain regions could be valuable, as the infiltrative nature of gliomas suggests a dependence on the GM or WM contrast of the specific region. This implies that the evaluated tumor CEST contrast values vary based on location.

Altogether, this study underscores the potential of CEST imaging as a robust and reliable tool for brain research, highlighting its clinical potential for detecting subtle changes and its sensitivity to regional and biological variability. Future studies, particularly in broader and diverse cohorts, could help refine CEST applications and confirm CEST utility across a range of neurological conditions.

## 5 Conclusion

This study identified significant regional variations in both relaxation‐compensated MTR_Rex_ and asymmetry‐based APTw CEST contrasts within the healthy human brain. Our findings underscore the importance of consistently placing ROIs in healthy‐appearing and region‐matched control tissue for relaxation‐compensated and APTw CEST, especially in cases where pathological conditions induce only subtle changes in CEST effects. These insights may contribute to a better understanding of APTw and relaxation‐compensated CEST contrasts in human brain tissues. Furthermore, they highlight the need for similar analyses focused on tumor tissue contrasts, particularly in relation to localization, to improve patient data evaluation and ultimately lead to more accurate diagnoses.

## Author Contributions

**Florian Kroh:** formal analysis, data curation, visualization, investigation, writing – original draft and review and editing. **Philip S. Boyd:** project administration, conceptualization, formal analysis, investigation, data curation, writing – review and editing. **Svenja Graß:** data curation, investigation, writing – review and editing. **Nikolaus von Knebel Doeberitz:** project administration, investigation, writing – review and editing. **Heinz‐Peter Schlemmer:** supervision, investigation, writing – review and editing. **Mark E. Ladd:** supervision, methodology, investigation, writing – review and editing. **Daniel Paech:** supervision, project administration, conceptualization, funding acquisition, investigation, writing – review and editing. **Andreas Korzowski:** supervision, project administration, methodology, conceptualization, investigation, writing – review and editing.

## Supporting information


**Figure S1:** Z‐spectra for gray matter (GM) (left) and white matter (WM) (right) from a healthy volunteer for a representative voxel in each of the evaluated regions (frontal [top], parietotemporal [top middle], occipital [bottom middle], calcarine sulcus [bottom]). Additionally, the four‐pool Lorentzian‐fit (black line) and the direct water saturation (blue), AMIDE (yellow), rNOE (red), and ssMT (green) Lorentz curves are shown. Besides slightly decreased ssMT contributions in the GM ROIs compared to WM, no obvious differences can be observed.

**Figure S2:** Boxplot showing the ROI analysis of a second representative volunteer as shown in Figure 4. The APTw (first row) and relaxation‐compensated CEST contrast values (MTR_Rex_, Rows 2–4) as well as T_1_ times (fifth row) are displayed for the GM (left column) and WM (middle column) ROIs located in the frontal lobe (FL, 1) parietotemporal region (ParTem, 2), occipital lobe (OC, 3) and the calcarine sulcus (Ccs, 4). Additionally, combined gray matter (GM) ROIs and combined white matter (WM) ROIs (right column) are displayed in the right column. A single asterisk (*) indicates that the marked group differs significantly (*p* < 0.05) from all other groups within the subfigure. Where an asterisk is followed by a group number (e.g., 2), the marked group differs significantly (*p* < 0.05) only from that specific group. This analysis matches well with the analysis of the volunteer shown in Figure 4.

**Figure S3:** Boxplot of the median signal values for the APTw (first row) and relaxation‐compensated MTR_Rex_ contrasts (Rows 2–4) as well as T_1_ times (fifth row) of all five female volunteers (25 ± 4.1 years, Table S1). Data are displayed for the GM (left column) and WM (middle column). ROIs located in the frontal lobe (FL) parietotemporal (ParTem), occipital lobe (OC), and the calcarine sulcus (Ccs). The combined gray matter (GM) ROIs and combined white matter (WM) ROIs (right column) are displayed in the right column.

**Figure S4:** Boxplot of the median signal values for the APTw (first row) and relaxation‐compensated MTR_Rex_ contrasts (Rows 2–4) as well as T_1_ times (fifth row) of all five male volunteers (25 ± 1.9 years; Table S2). Data are displayed for the GM (left column) and WM (middle column). ROIs located in the frontal lobe (FL) parietotemporal (ParTem), occipital lobe (OC), and the calcarine sulcus (Ccs). The combined gray matter (GM) ROIs and combined white matter (WM) ROIs (right column) are displayed in the right column.

**Figure S5:** The same two slices as displayed in Figures 1A and 3 are shown for APTw (A); fluid‐suppressed APTw (B); the Lorentzian difference (LD; C) AMIDE, rNOE, and ssMT; and the relaxation‐compensated AREX (D) AMIDE, rNOE, and ssMT to present the visual characteristics of the analyzed contrasts. The APTw and fluid‐suppressed APTw show clear differences between GM and WM with an increased GM contrast. Furthermore, vascular signals are suppressed in the fluid‐suppressed APTw contrast. In contrast, the AREX rNOE and ssMT show a opposite and T_1_‐like behavior with a clearly increased WM contrast when compared to GM. Lastly, the AREX AMIDE GM‐WM contrast behavior is observably different to the MTR_Rrex_ AMIDE contrasts as it is no longer showing a clear hyperintense GM region and instead a slightly increased contrast in WM. Notably, the fluid‐suppressed APTw contrast maps depict similar ringing artifacts in the parieto‐occipital regions as the APTw contrast. Finally, the LD contrasts show similar characteristics than the MTR_Rex_ contrasts in Figure 3.

**Figure S6:** Boxplot of the median signal values for the APTw _fluidsupp_ (first row) and relaxation‐compensated AREX contrasts (Rows 2–4) of the 10 volunteers (25 ± 3.1 years, five female). Data are displayed for the GM (left column) and WM (middle column). ROIs located in the frontal lobe (FL, 1) parietotemporal (ParTem, 2), occipital lobe (OC, 3), and the calcarine sulcus (Ccs, 4). The combined gray matter (GM) ROIs and combined white matter (WM) ROIs (right column) are displayed in the right column. A single asterisk (*) indicates that the marked group differs significantly (*p* < 0.05) from all other groups of ROIs within the subfigure. Where an asterisk is followed by a group number (e.g., 2), the marked group differs significantly (*p* < 0.05) only from that specific group.

**Figure S7:** Boxplot of the median signal values for the LD contrasts (Rows 2–4) of the 10 volunteers (25 ± 3.1 years, five female). Data are displayed for the GM (left column) and WM (middle column). ROIs located in the frontal lobe (FL, 1) parietotemporal (ParTem, 2), occipital lobe (OC, 3), and the calcarine sulcus (Ccs, 4). The combined gray matter (GM) ROIs and combined white matter (WM) ROIs (right column) are displayed in the right column. A single asterisk (*) indicates that the marked group differs significantly (*p* < 0.05) from all other groups of ROIs within the subfigure. Where an asterisk is followed by a group number (e.g., 2), the marked group differs significantly (*p* < 0.05) only from that specific group.

**Table S1:** Summary of the median ROI value analysis for the GM ROIs across all five male (25 ± 1.9 years) and female (25 ± 4.1 years) volunteers. Mean and standard deviation were calculated for APTw, MTR_Rex_ AMIDE, MTR_Rex_ rNOE, MTR_Rex_ ssMT, and the quantitative T_1_ measurement in the combined GM ROI as well as for the individual regions (FL = frontal lobe, ParTem = parietotemporal lobe, OC = occipital lobe, Ccs = calcarine sulcus).

**Table S2:** Summary of the median ROI value analysis for the WM ROIs across all five male (25 ± 1.9 years) and female (25 ± 4.1 years) volunteers. Mean and standard deviation were calculated for APTw, MTR_Rex_ AMIDE, MTR_Rex_ rNOE, MTR_Rex_ ssMT, and the quantitative T_1_ measurement in the combined WM ROI as well as for the individual regions (FL = frontal lobe, ParTem = parietotemporal lobe, OC = occipital lobe, Ccs = calcarine sulcus).

**Table S3:** Summary of the median ROI value analysis across all 10 volunteers. Mean and standard deviation (SD) were calculated for APTw,fs, AREX AMIDE, AREX rNOE, AREX ssMT, LD AMIDE, LD rNOE, and LD ssMT and in the combined GM and WM ROIs as well as for the individual regions (FL = frontal lobe, ParTem = parietotemporal lobe, OC = occipital lobe, Ccs = calcarine sulcus).

**Table S4:** Summary of the median ROI value analysis across all 10 volunteers. Coefficients of variation (CVs) were calculated for MTR_Rex_ AMIDE, MTR_Rex_ rNOE, MTR_Rex_ ssMT, T_1_, AREX AMIDE, AREX rNOE, AREX ssMT, LD AMIDE, LD rNOE, and LD ssMT and in the combined GM and WM ROIs as well as for the individual regions (FL = frontal lobe, ParTem = parietotemporal lobe, OC = occipital lobe, Ccs = calcarine sulcus). They were not calculated for the APTw contrasts since these metrics are defined on an interval scale.

**Data S1:** Supplementary information.

## Data Availability

The data that support the findings of this study are available from the corresponding author upon reasonable request.

## References

[1] S. Forsén , R. A. Hoffman , S. Forsén , and R. A. Hoffman , “Study of Moderately Rapid Chemical Exchange Reactions by Means of Nuclear Magnetic Double Resonance,” JChPh 39, no. 11 (1963): 2892–2901, 10.1063/1.1734121.

[2] K. M. Ward , A. H. Aletras , and R. S. Balaban , “A New Class of Contrast Agents for MRI Based on Proton Chemical Exchange Dependent Saturation Transfer (CEST),” Journal of Magnetic Resonance 143, no. 1 (2000): 79–87, 10.1006/JMRE.1999.1956. 10698648

[3] J. Zhou , J. F. Payen , D. A. Wilson , R. J. Traystman , and P. C. M. Van Zijl , “Using the Amide Proton Signals of Intracellular Proteins and Peptides to Detect pH Effects in MRI,” Nature Medicine 9, no. 8 (2003): 1085–1090, 10.1038/NM907. 12872167

[4] P. C. M. Van Zijl and N. N. Yadav , “Chemical Exchange Saturation Transfer (CEST): What Is in a Name and What Isn't?,” Magnetic Resonance in Medicine 65, no. 4 (2011): 927–948, 10.1002/MRM.22761. 21337419 PMC3148076

[5] D. Paech , J. Windschuh , J. Oberhollenzer , et al., “Assessing the Predictability of IDH Mutation and MGMT Methylation Status in Glioma Patients Using Relaxation‐Compensated Multipool CEST MRI at 7.0 T,” Neuro‐Oncology 20, no. 12 (2018): 1661–1671, 10.1093/NEUONC/NOY073. 29733378 PMC6231210

[6] S. Jiang , T. Zou , C. G. Eberhart , et al., “Predicting IDH Mutation Status in Grade II Gliomas Using Amide Proton Transfer‐Weighted (APTw) MRI,” Magnetic Resonance in Medicine 78, no. 3 (2017): 1100–1109, 10.1002/MRM.26820. 28714279 PMC5561497

[7] O. Togao , T. Yoshiura , J. Keupp , et al., “Amide Proton Transfer Imaging of Adult Diffuse Gliomas: Correlation With Histopathological Grades,” Neuro‐Oncology 16, no. 3 (2014): 441–448, 10.1093/NEUONC/NOT158. 24305718 PMC3922507

[8] J. E. Meissner , A. Korzowski , S. Regnery , et al., “Early Response Assessment of Glioma Patients to Definitive Chemoradiotherapy Using Chemical Exchange Saturation Transfer Imaging at 7 T,” Journal of Magnetic Resonance Imaging. 50, no. 4 (2019): 1268–1277, 10.1002/JMRI.26702. 30864193

[9] H. Mehrabian , S. Myrehaug , H. Soliman , A. Sahgal , and G. J. Stanisz , “Evaluation of Glioblastoma Response to Therapy With Chemical Exchange Saturation Transfer,” International Journal of Radiation Oncology, Biology, Physics 101, no. 3 (2018): 713–723, 10.1016/J.IJROBP.2018.03.057. 29893279

[10] H. Mehrabian , K. L. Desmond , H. Soliman , A. Sahgal , and G. J. Stanisz , “Differentiation Between Radiation Necrosis and Tumor Progression Using Chemical Exchange Saturation Transfer,” Clinical Cancer Research. 23, no. 14 (2017): 3667–3675, 10.1158/1078-0432.CCR-16-2265. 28096269

[11] F. Kroh , N. von Knebel Doeberitz , J. Breitling , et al., “Semi‐Solid MT and APTw CEST‐MRI Predict Clinical Outcome of Patients With Glioma Early After Radiotherapy,” Magnetic Resonance in Medicine 90, no. 4 (2023): 1569–1581, 10.1002/MRM.29746. 37317562

[12] K. Karimian‐Jazi , N. Enbergs , E. Golubtsov , et al., “Differentiating Glioma Recurrence and Pseudoprogression by APTw CEST MRI,” Investigative Radiology 60 (2024): 414–422, 10.1097/RLI.0000000000001145. 39644107

[13] D. Paech , C. Dreher , S. Regnery , et al., “Relaxation‐Compensated Amide Proton Transfer (APT) MRI Signal Intensity Is Associated With Survival and Progression in High‐Grade Glioma Patients,” European Radiology 29, no. 9 (2019): 4957–4967, 10.1007/S00330-019-06066-2/METRICS. 30809720

[14] N. von Knebel Doeberitz , F. Kroh , J. Breitling , et al., “CEST Imaging of the APT and ssMT Predict the Overall Survival of Patients with Glioma at the First Follow‐Up After Completion of Radiotherapy at 3T,” Radiotherapy and Oncology 184 (2023): 109694, 10.1016/J.RADONC.2023.109694. 37150450

[15] M. Haris , K. Nath , K. Cai , et al., “Imaging of Glutamate Neurotransmitter Alterations in Alzheimer's Disease,” NMR in Biomedicine 26, no. 4 (2013): 386–391, 10.1002/NBM.2875. 23045158 PMC3556355

[16] C. Li , S. Peng , R. Wang , et al., “Chemical Exchange Saturation Transfer MR Imaging of Parkinson's Disease at 3 Tesla,” European Radiology 24, no. 10 (2014): 2631–2639, 10.1007/S00330-014-3241-7. 25038850 PMC4471479

[17] M. Wang , X. Hong , C. F. Chang , et al., “Simultaneous Detection and Separation of Hyperacute Intracerebral Hemorrhage and Cerebral Ischemia Using Amide Proton Transfer MRI,” Magnetic Resonance in Medicine 74, no. 1 (2015): 42–50, 10.1002/MRM.25690. 25879165 PMC4608848

[18] J. Zhou , M. Zaiss , L. Knutsson , et al., “Review and Consensus Recommendations on Clinical APT‐Weighted Imaging Approaches at 3T: Application to Brain Tumors,” Magnetic Resonance in Medicine 88, no. 2 (2022): 546–574, 10.1002/MRM.29241. 35452155 PMC9321891

[19] M. Zaiss , J. Windschuh , S. Goerke , et al., “Downfield‐NOE‐Suppressed Amide‐CEST‐MRI at 7 Tesla Provides a Unique Contrast in Human Glioblastoma,” Magnetic Resonance in Medicine 77, no. 1 (2017): 196–208, 10.1002/MRM.26100/ASSET/SUPINFO/MRM26100-SUP-0001-SUPPINFO.DOCX. 26845067

[20] S. Goerke , Y. Soehngen , A. Deshmane , et al., “Relaxation‐Compensated APT and rNOE CEST‐MRI of Human Brain Tumors at 3 T,” Magnetic Resonance in Medicine 82, no. 2 (2019): 622–632, 10.1002/MRM.27751. 30927313

[21] S. Goerke , J. Breitling , A. Korzowski , et al., “Clinical Routine Acquisition Protocol for 3D Relaxation‐Compensated APT and rNOE CEST‐MRI of the Human Brain at 3T,” Magnetic Resonance in Medicine 86, no. 1 (2021): 393–404, 10.1002/MRM.28699. 33586217

[22] A. E. Cronin , P. Liebig , S. A. Detombe , N. Duggal , and R. Bartha , “Reproducibility of 3D Chemical Exchange Saturation Transfer (CEST) Contrasts in the Healthy Brain at 3T,” Scientific Reports 14, no. 1 (2024): 1–13, 10.1038/s41598-024-75777-4. 39465319 PMC11514173

[23] J. Ashburner and K. J. Friston , “Unified Segmentation,” NeuroImage 26, no. 3 (2005): 839–851, 10.1016/J.NEUROIMAGE.2005.02.018. 15955494

[24] E. T. Rolls , C. C. Huang , C. P. Lin , J. Feng , and M. Joliot , “Automated Anatomical Labelling Atlas 3,” Neuroimage 206 (2020): 116189, 10.1016/J.NEUROIMAGE.2019.116189. 31521825

[25] M. Zaiss , P. Ehses , and K. Scheffler , “Snapshot‐CEST: Optimizing Spiral‐Centric‐Reordered Gradient Echo Acquisition for Fast and Robust 3D CEST MRI at 9.4 T,” NMR in Biomedicine 31, no. 4 (2018): e3879, 10.1002/NBM.3879. 29372571

[26] A. Deshmane , M. Zaiss , T. Lindig , et al., “3D Gradient Echo Snapshot CEST MRI With Low Power Saturation for Human Studies at 3T,” Magnetic Resonance in Medicine 81, no. 4 (2019): 2412–2423, 10.1002/MRM.27569. 30431179 PMC6718050

[27] P. Schuenke , J. Windschuh , V. Roeloffs , M. E. Ladd , P. Bachert , and M. Zaiss , “Simultaneous Mapping of Water Shift and B1(WASABI)—Application to Field‐Inhomogeneity Correction of CEST MRI Data,” Magnetic Resonance in Medicine 77, no. 2 (2017): 571–580, 10.1002/MRM.26133. 26857219

[28] M. A. Bernstein , K. F. King , and X. J. Zhou , Handbook of MRI Pulse Sequences. Handbook of MRI Pulse Sequences (Academic Press, 2004), 1–1017, 10.1016/B978-0-12-092861-3.X5000-6.

[29] J. Zhou , H. Y. Heo , L. Knutsson , P. C. M. van Zijl , and S. Jiang , “APT‐Weighted MRI: Techniques, Current Neuro Applications, and Challenging Issues,” Journal of Magnetic Resonance Imaging. 50, no. 2 (2019): 347–364, 10.1002/JMRI.26645. 30663162 PMC6625919

[30] M. Nolden , S. Zelzer , A. Seitel , et al., “The Medical Imaging Interaction Toolkit: Challenges and Advances: 10 Years of Open‐Source Development,” International Journal of Computer Assisted Radiology and Surgery 8, no. 4 (2013): 607–620, 10.1007/S11548-013-0840-8. 23588509

[31] J. Breitling , A. Korzowski , N. Kempa , et al., “Motion Correction for Three‐Dimensional Chemical Exchange Saturation Transfer Imaging Without Direct Water Saturation Artifacts,” NMR in Biomedicine 35, no. 7 (2022): e4720, 10.1002/NBM.4720. 35233847

[32] J. R. Schüre , S. Casagranda , M. Sedykh , et al., “Fluid Suppression in Amide Proton Transfer‐Weighted (APTw) CEST Imaging: New Theoretical Insights and Clinical Benefits,” Magnetic Resonance in Medicine 91, no. 4 (2024): 1354–1367, 10.1002/MRM.29915. 38073061

[33] J. Breitling , A. Deshmane , S. Goerke , et al., “Adaptive Denoising for Chemical Exchange Saturation Transfer MR Imaging,” NMR in Biomedicine 32, no. 11 (2019): e4133, 10.1002/NBM.4133. 31361064

[34] M. Zaiss and P. Bachert , “Chemical Exchange Saturation Transfer (CEST) and MR Z‐Spectroscopy in Vivo: A Review of Theoretical Approaches and Methods,” Physics in Medicine and Biology 58, no. 22 (2013): R221–R269, 10.1088/0031-9155/58/22/R221. 24201125

[35] M. Zaiss , J. Xu , S. Goerke , et al., “Inverse Z‐Spectrum Analysis for Spillover‐, MT‐, and T1‐Corrected Steady‐State Pulsed CEST‐MRI ‐ Application to pH‐Weighted MRI of Acute Stroke,” NMR in Biomedicine 27, no. 3 (2014): 240–252, 10.1002/NBM.3054. 24395553 PMC4520220

[36] J. Windschuh , M. Zaiss , J. E. Meissner , et al., “Correction of B1‐Inhomogeneities for Relaxation‐Compensated CEST Imaging at 7T,” NMR in Biomedicine 28, no. 5 (2015): 529–537, 10.1002/NBM.3283. 25788155

[37] J. B. Lee , J. E. Park , S. C. Jung , et al., “Repeatability of Amide Proton Transfer–Weighted Signals in the Brain According to Clinical Condition and Anatomical Location,” European Radiology 30, no. 1 (2020): 346–356, 10.1007/S00330-019-06285-7/METRICS. 31338651

[38] I. Pflüger , A. Rastogi , S. Casagranda , et al., “Amide Proton Transfer Weighted MRI Measurements Yield Consistent and Repeatable Results in Patients With Gliomas: A Prospective Test‐Retest Study,” European Radiology 35 (2024): 3367–3379, 10.1007/S00330-024-11197-2. 39694884

[39] M. N. Voelker , O. Kraff , S. Goerke , et al., “The Traveling Heads 2.0: Multicenter Reproducibility of Quantitative Imaging Methods at 7 Tesla,” NeuroImage 232 (2021): 117910, 10.1016/J.NEUROIMAGE.2021.117910. 33647497

[40] W. G. Murphy , “The Sex Difference in Haemoglobin Levels in Adults — Mechanisms, Causes, and Consequences,” Blood Reviews 28, no. 2 (2014): 41–47, 10.1016/J.BLRE.2013.12.003. 24491804

[41] A. Mennecke , K. M. Khakzar , A. German , et al., “7 Tricks for 7 T CEST: Improving the Reproducibility of Multipool Evaluation Provides Insights Into the Effects of Age and the Early Stages of Parkinson's Disease,” NMR in Biomedicine 36, no. 6 (2023): e4717, 10.1002/NBM.4717. 35194865

[42] C. K. Jones , A. Huang , J. Xu , et al., “Nuclear Overhauser Enhancement (NOE) Imaging in the Human Brain at 7T,” NeuroImage 77 (2013): 114–124, 10.1016/J.NEUROIMAGE.2013.03.047. 23567889 PMC3848060

[43] X. Xu , N. N. Yadav , H. Zeng , et al., “Magnetization Transfer Contrast‐Suppressed Imaging of Amide Proton Transfer and Relayed Nuclear Overhauser Enhancement Chemical Exchange Saturation Transfer Effects in the Human Brain at 7T,” Magnetic Resonance in Medicine 75, no. 1 (2016): 88–96, 10.1002/mrm.25990. 26445350 PMC4715767

[44] T. Jin , P. Wang , X. Zong , and S. G. Kim , “MR Imaging of the Amide‐Proton Transfer Effect and the pH‐Insensitive Nuclear Overhauser Effect at 9.4 T,” Magnetic Resonance in Medicine 69, no. 3 (2013): 760–770, 10.1002/mrm.24315. 22577042 PMC3419318

[45] J. S. O'Brien and E. L. Sampson , “Lipid Composition of the Normal Human Brain: Gray Matter, White Matter, and Myelin,” Journal of Lipid Research 6, no. 4 (1965): 537–544, 10.1016/s0022-2275(20)39619-x. 5865382

[46] P. F. M. Ribeiro , L. Ventura‐Antunes , M. Gabi , et al., “The Human Cerebral Cortex Is Neither One nor Many: Neuronal Distribution Reveals Two Quantitatively Different Zones in the Gray Matter, Three in the White Matter, and Explains Local Variations in Cortical Folding,” Frontiers in Neuroanatomy 7, no. SEP (2013): 28, 10.3389/FNANA.2013.00028/ABSTRACT. 24032005 PMC3759024

[47] J. Platek , F. Kroh , V. L. Franke , et al., (ISMRM 2023) Analysis of Regional Differences in pH Values Obtained via 31P MRSI at 7T. June 2023. Accessed January 29, 2025, https://archive.ismrm.org/2023/4731.html.

[48] Keupp, J. , and O. Togao . ISMRM 2018. Magnetization Transfer Ratio Based Metric for APTw or CESTw MRI Suppressing Signal From Fluid Compartments ‐ Initial Application to Glioblastoma Assessment. 2018. Accessed January 29, 2025, https://archive.ismrm.org/2018/3156.html.

[49] L. Mancini , S. Casagranda , G. Gautier , et al., “CEST MRI Provides Amide/Amine Surrogate Biomarkers for Treatment‐Naïve Glioma sub‐Typing,” European Journal of Nuclear Medicine and Molecular Imaging 49, no. 7 (2022): 2377–2391, 10.1007/S00259-022-05676-1. 35029738 PMC9165287

[50] M. Knutsson , T. Salomonsson , F. Durmo , et al., “Differentiation Between Glioblastoma and Solitary Brain Metastases Using Perfusion and Amide Proton Transfer Weighted MRI,” Frontiers in Neuroscience 19 (2025): 1533799, 10.3389/FNINS.2025.1533799/BIBTEX. 39975970 PMC11836003

[51] F. Durmo , A. Rydhog , F. Testud , et al., “Assessment of Amide Proton Transfer Weighted (APTw) MRI for Pre‐Surgical Prediction of Final Diagnosis in Gliomas,” PLoS ONE 15, no. 12 (2020): e0244003, 10.1371/JOURNAL.PONE.0244003. 33373375 PMC7771875

