# Supplementary figures and images for: CEST Contrasts Exhibit Significant Regional Variations in the Human Brain at 3 T
Source: NMR Biomed. 2025 Nov 13;38(12):e70177. doi: 10.1002/nbm.70177 (PMC12613240; doi:10.1002/nbm.70177)

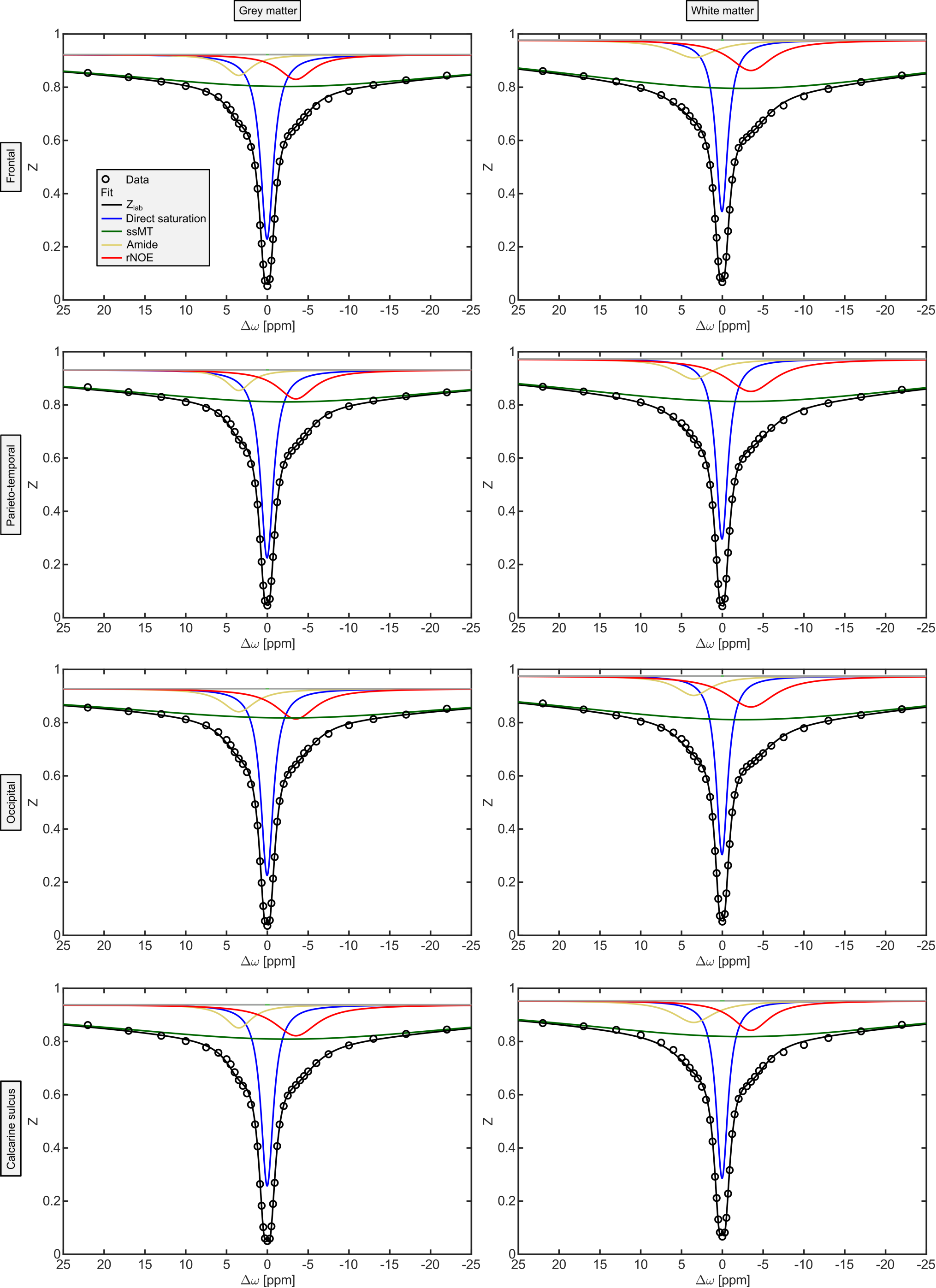

Supplement: Supplementary file 1 — Figure S1: Z‐spectra for gray matter (GM) (left) and white matter (WM) (right) from a healthy volunteer for a representative voxel in each of the evaluated regions (frontal [top], parietotemporal [top middle], occipital [bottom middle], calcarine sulcus [bottom]). Additionally, the four‐pool Lorentzian‐fit (black line) and the direct water saturation (blue), AMIDE (yellow), rNOE (red), and ssMT (green) Lorentz curves are shown. Besides slightly decreased ssMT contributions in the GM ROIs compared to WM, no obvious differences can be observed. [file NBM-38-e70177-s005.tiff]

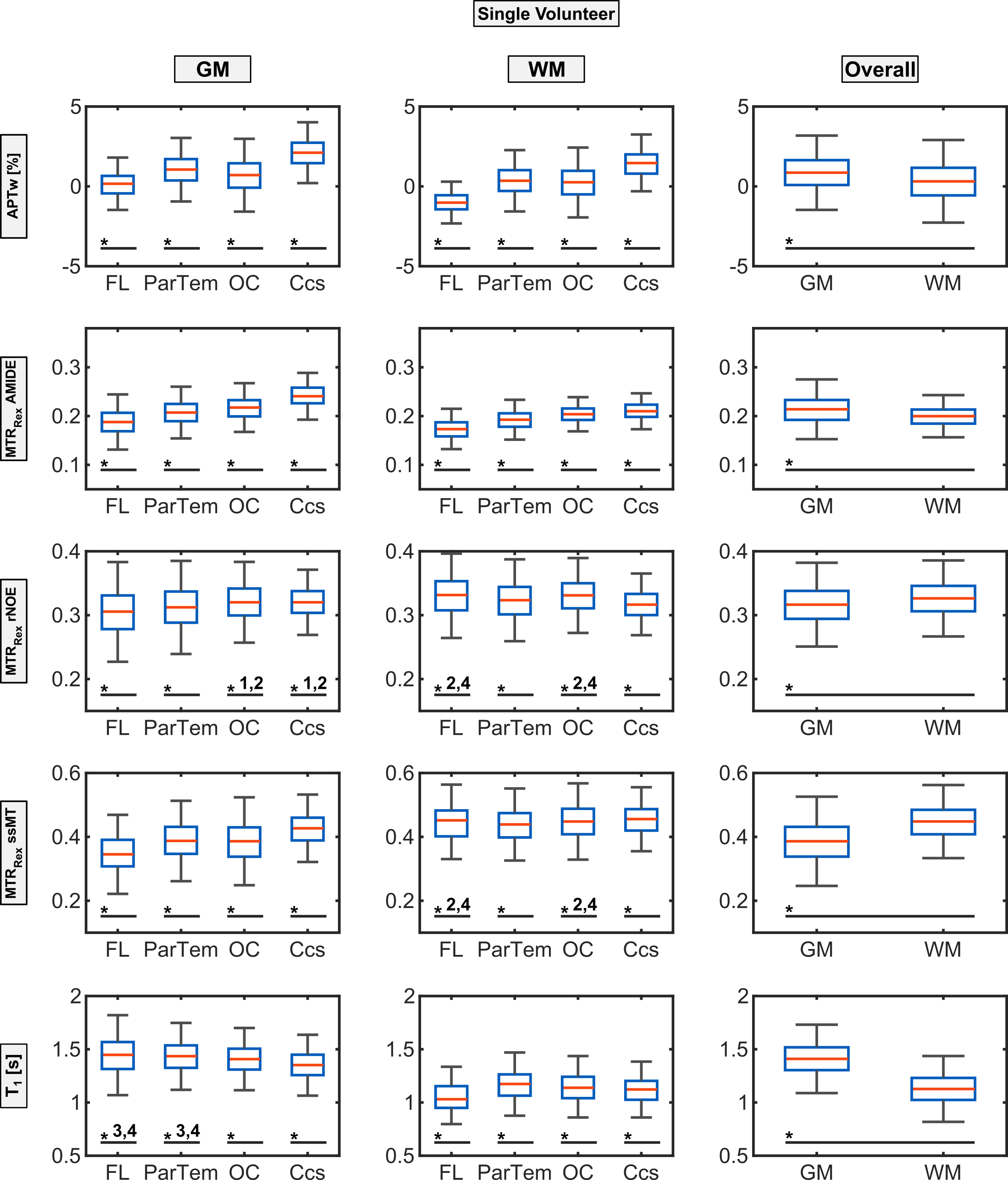

Supplement: Supplementary file 2 — Figure S2: Boxplot showing the ROI analysis of a second representative volunteer as shown in Figure 4. The APTw (first row) and relaxation‐compensated CEST contrast values (MTRRex, Rows 2–4) as well as T1 times (fifth row) are displayed for the GM (left column) and WM (middle column) ROIs located in the frontal lobe (FL, 1) parietotemporal region (ParTem, 2), occipital lobe (OC, 3) and the calcarine sulcus (Ccs, 4). Additionally, combined gray matter (GM) ROIs and combined white matter (WM) ROIs (right column) are displayed in the right column. A single asterisk (*) indicates that the marked group differs significantly (p < 0.05) from all other groups within the subfigure. Where an asterisk is followed by a group number (e.g., 2), the marked group differs significantly (p < 0.05) only from that specific group. This analysis matches well with the analysis of the volunteer shown in Figure 4. [file NBM-38-e70177-s003.tiff]

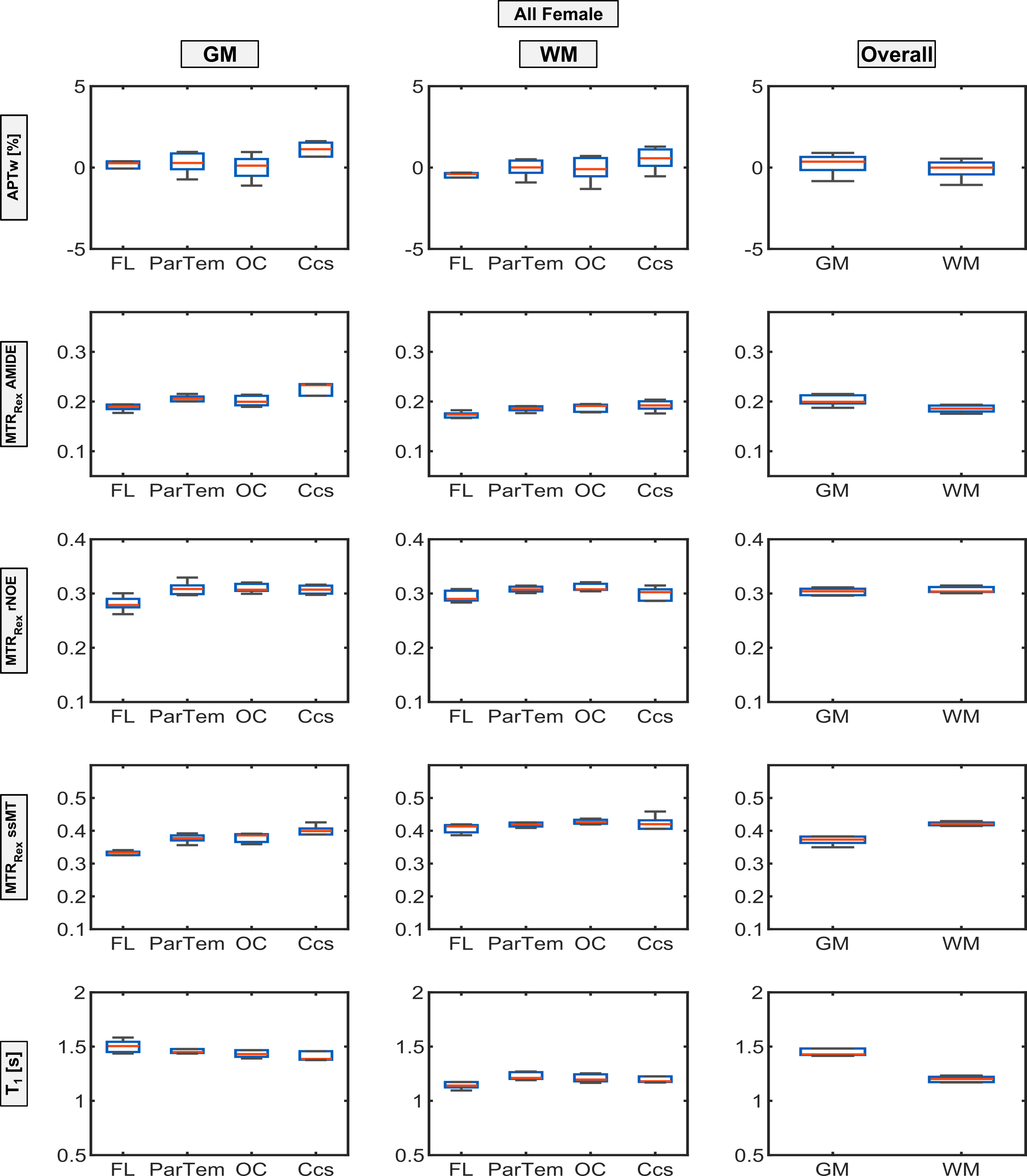

Supplement: Supplementary file 3 — Figure S3: Boxplot of the median signal values for the APTw (first row) and relaxation‐compensated MTRRex contrasts (Rows 2–4) as well as T1 times (fifth row) of all five female volunteers (25 ± 4.1 years, Table S1). Data are displayed for the GM (left column) and WM (middle column). ROIs located in the frontal lobe (FL) parietotemporal (ParTem), occipital lobe (OC), and the calcarine sulcus (Ccs). The combined gray matter (GM) ROIs and combined white matter (WM) ROIs (right column) are displayed in the right column. [file NBM-38-e70177-s006.tiff]

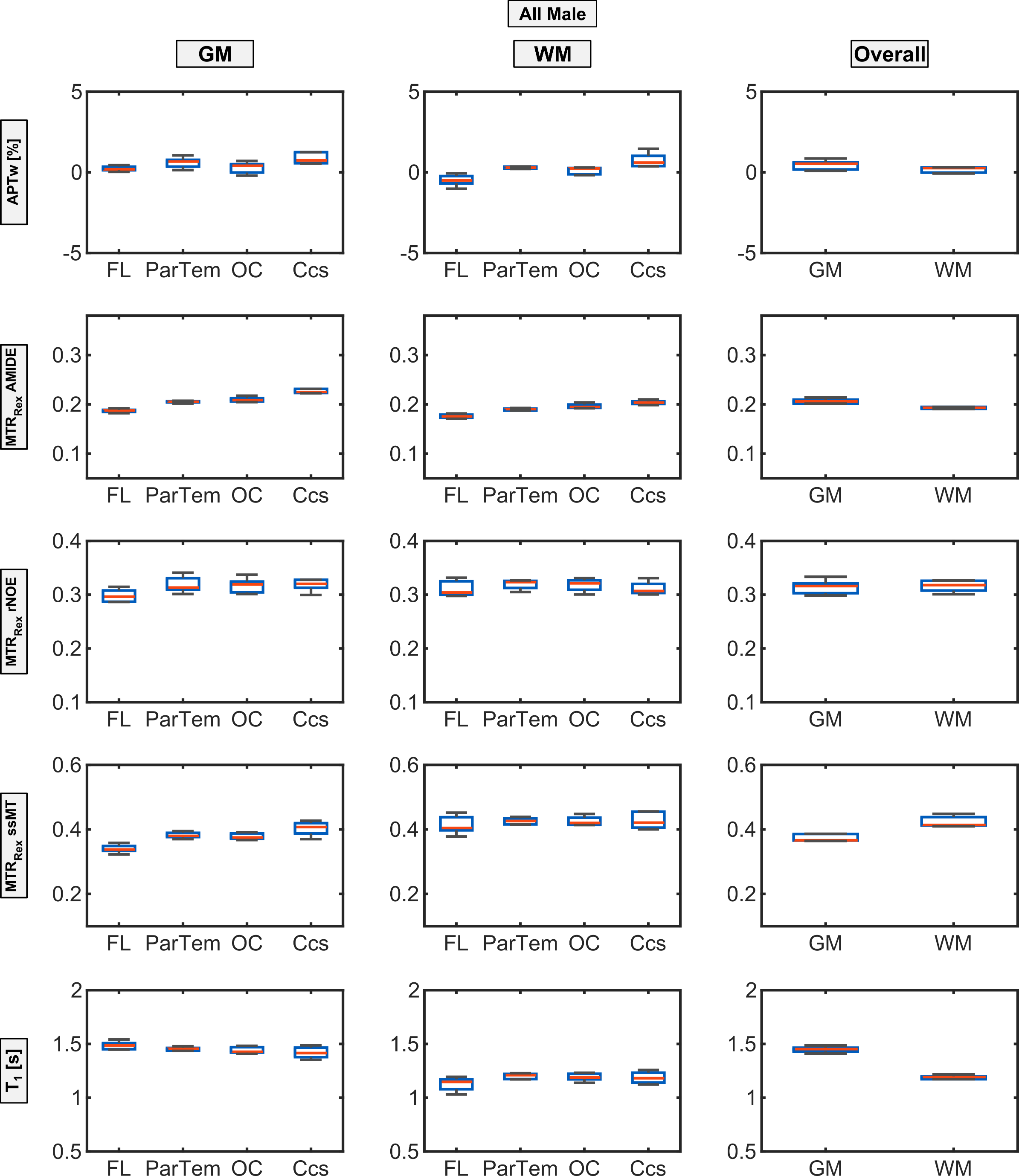

Supplement: Supplementary file 4 — Figure S4: Boxplot of the median signal values for the APTw (first row) and relaxation‐compensated MTRRex contrasts (Rows 2–4) as well as T1 times (fifth row) of all five male volunteers (25 ± 1.9 years; Table S2). Data are displayed for the GM (left column) and WM (middle column). ROIs located in the frontal lobe (FL) parietotemporal (ParTem), occipital lobe (OC), and the calcarine sulcus (Ccs). The combined gray matter (GM) ROIs and combined white matter (WM) ROIs (right column) are displayed in the right column. [file NBM-38-e70177-s009.tiff]

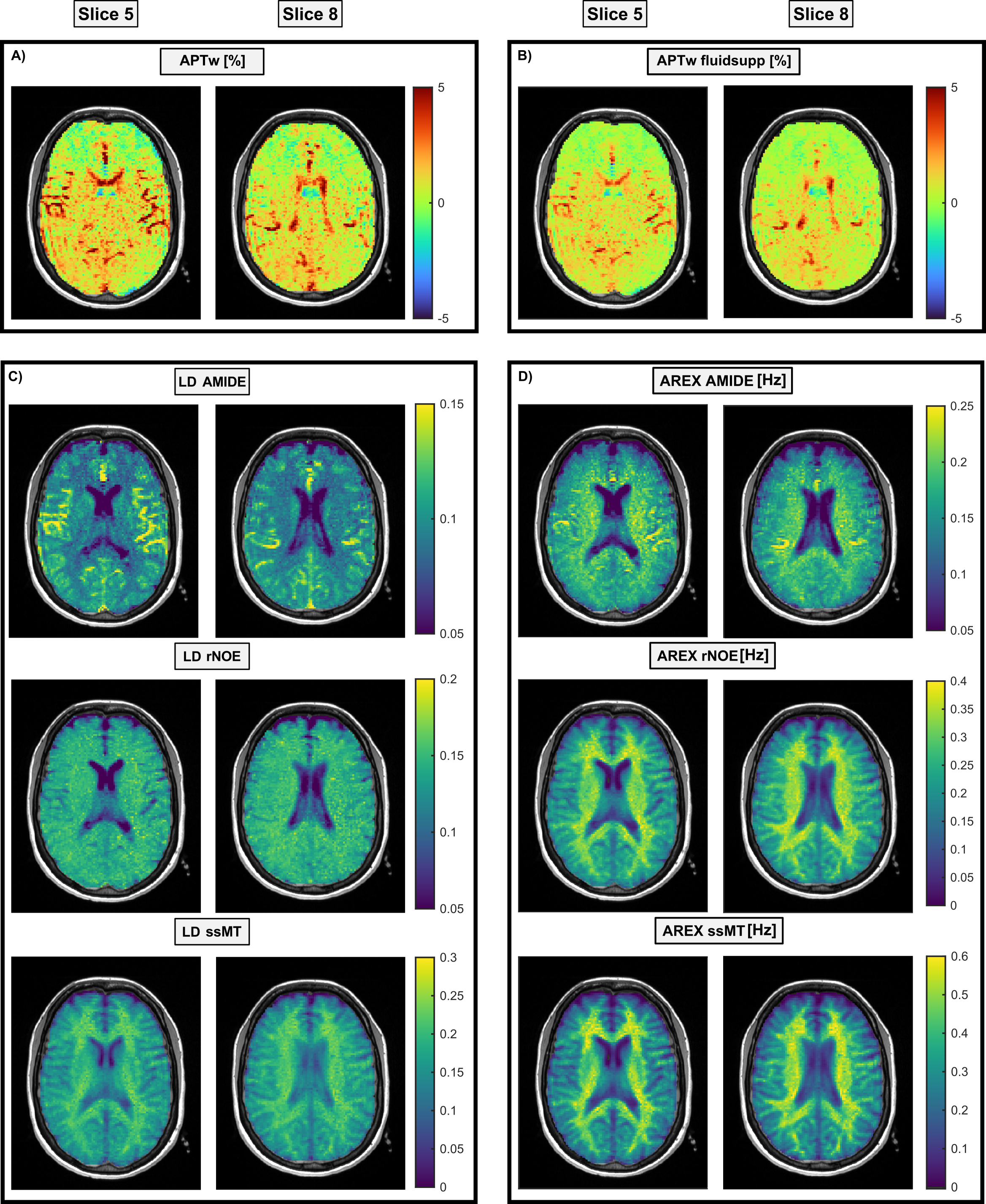

Supplement: Supplementary file 5 — Figure S5: The same two slices as displayed in Figures 1A and 3 are shown for APTw (A); fluid‐suppressed APTw (B); the Lorentzian difference (LD; C) AMIDE, rNOE, and ssMT; and the relaxation‐compensated AREX (D) AMIDE, rNOE, and ssMT to present the visual characteristics of the analyzed contrasts. The APTw and fluid‐suppressed APTw show clear differences between GM and WM with an increased GM contrast. Furthermore, vascular signals are suppressed in the fluid‐suppressed APTw contrast. In contrast, the AREX rNOE and ssMT show a opposite and T1‐like behavior with a clearly increased WM contrast when compared to GM. Lastly, the AREX AMIDE GM‐WM contrast behavior is observably different to the MTRRrex AMIDE contrasts as it is no longer showing a clear hyperintense GM region and instead a slightly increased contrast in WM. Notably, the fluid‐suppressed APTw contrast maps depict similar ringing artifacts in the parieto‐occipital regions as the APTw contrast. Finally, the LD contrasts show similar characteristics than the MTRRex contrasts in Figure 3. [file NBM-38-e70177-s007.tiff]

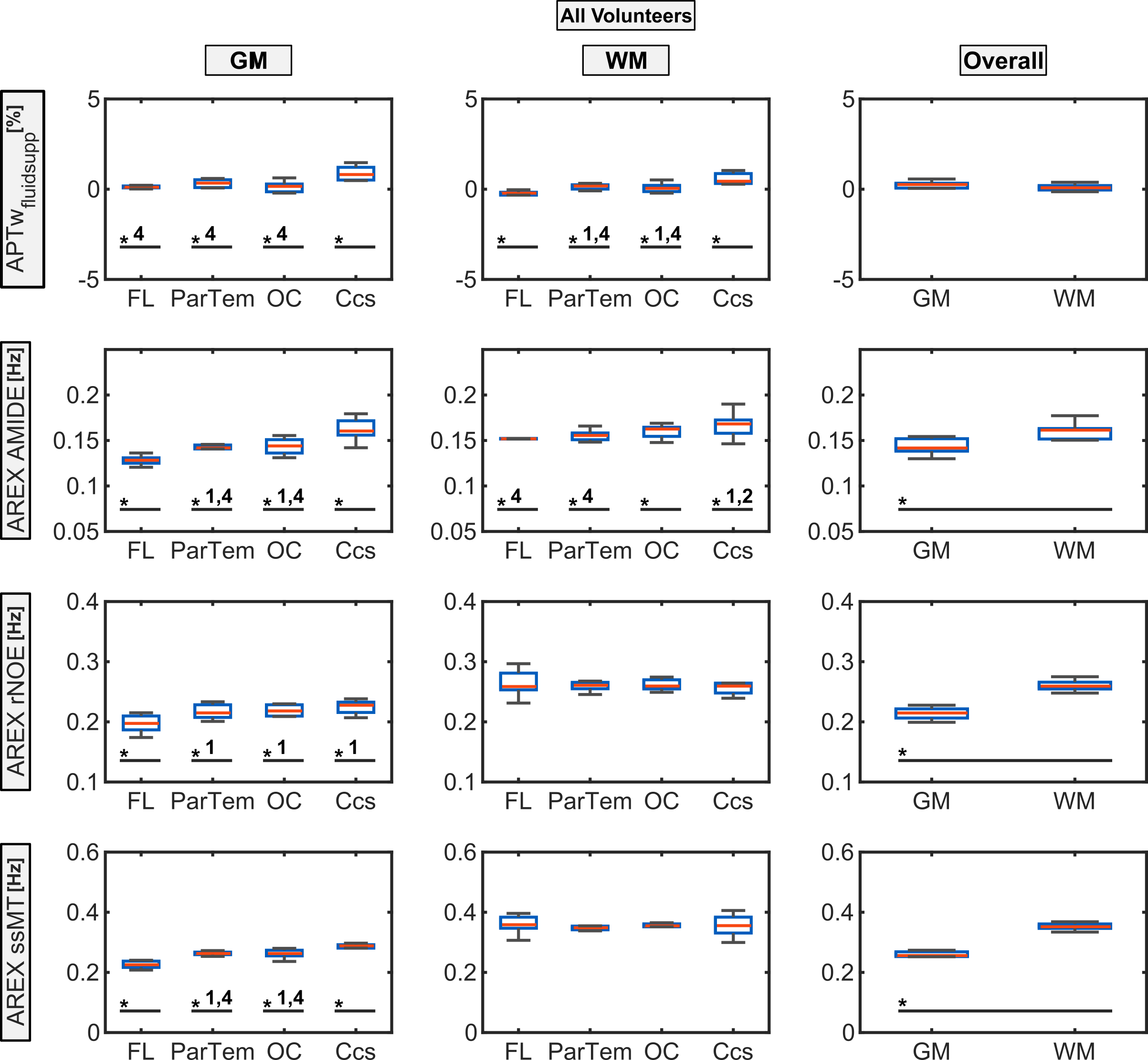

Supplement: Supplementary file 6 — Figure S6: Boxplot of the median signal values for the APTw fluidsupp (first row) and relaxation‐compensated AREX contrasts (Rows 2–4) of the 10 volunteers (25 ± 3.1 years, five female). Data are displayed for the GM (left column) and WM (middle column). ROIs located in the frontal lobe (FL, 1) parietotemporal (ParTem, 2), occipital lobe (OC, 3), and the calcarine sulcus (Ccs, 4). The combined gray matter (GM) ROIs and combined white matter (WM) ROIs (right column) are displayed in the right column. A single asterisk (*) indicates that the marked group differs significantly (p < 0.05) from all other groups of ROIs within the subfigure. Where an asterisk is followed by a group number (e.g., 2), the marked group differs significantly (p < 0.05) only from that specific group. [file NBM-38-e70177-s001.tiff]

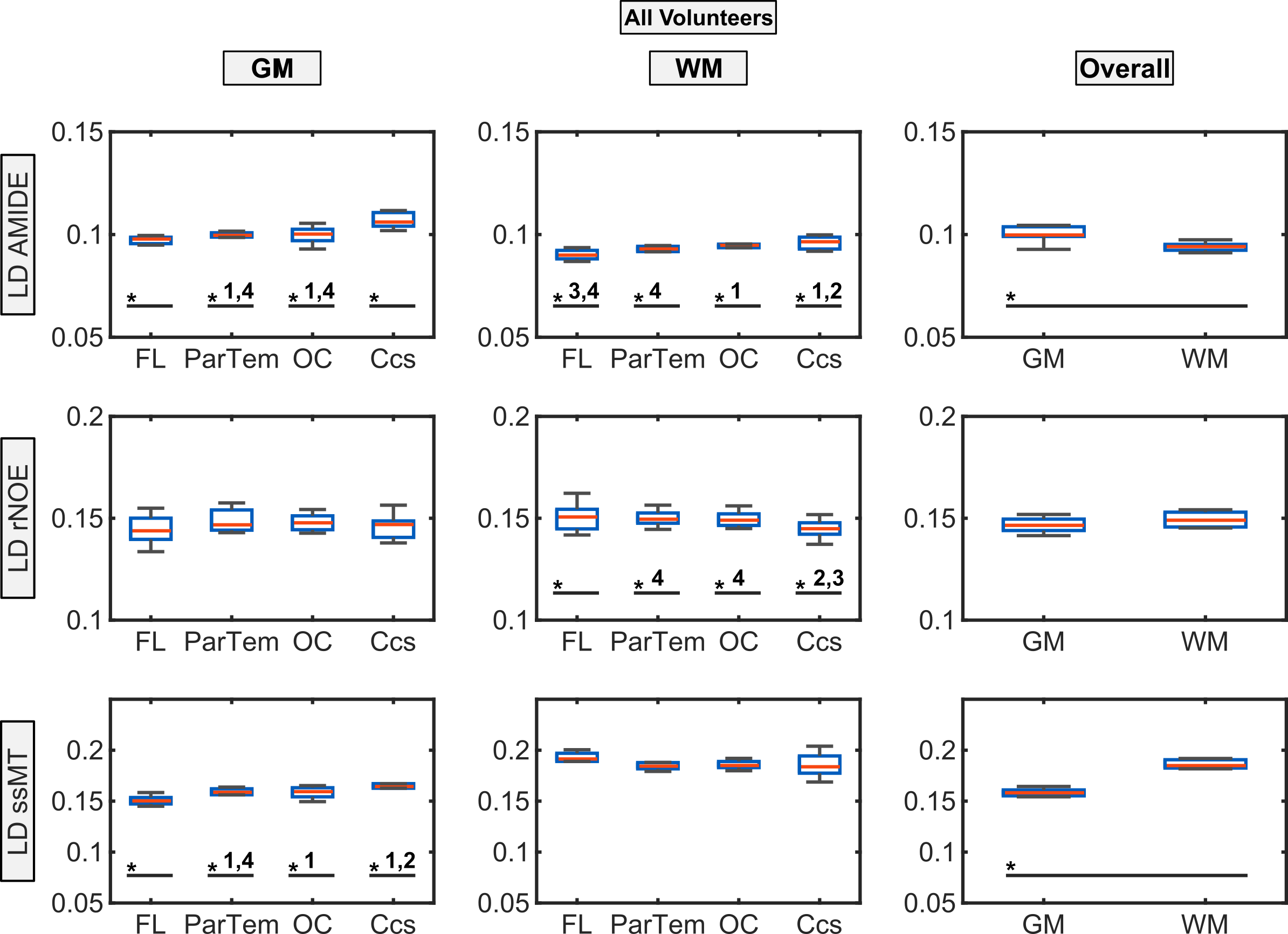

Supplement: Supplementary file 7 — Figure S7: Boxplot of the median signal values for the LD contrasts (Rows 2–4) of the 10 volunteers (25 ± 3.1 years, five female). Data are displayed for the GM (left column) and WM (middle column). ROIs located in the frontal lobe (FL, 1) parietotemporal (ParTem, 2), occipital lobe (OC, 3), and the calcarine sulcus (Ccs, 4). The combined gray matter (GM) ROIs and combined white matter (WM) ROIs (right column) are displayed in the right column. A single asterisk (*) indicates that the marked group differs significantly (p < 0.05) from all other groups of ROIs within the subfigure. Where an asterisk is followed by a group number (e.g., 2), the marked group differs significantly (p < 0.05) only from that specific group. [file NBM-38-e70177-s004.tiff]
