# Supplementary material for: CEST Contrasts Exhibit Significant Regional Variations in the Human Brain at 3 T
Source: NMR Biomed. 2025 Nov 13;38(12):e70177. doi: 10.1002/nbm.70177 (PMC12613240; doi:10.1002/nbm.70177)
Supplement: Supplementary file 8 — Table S1: Summary of the median ROI value analysis for the GM ROIs across all five male (25 ± 1.9 years) and female (25 ± 4.1 years) volunteers. Mean and standard deviation were calculated for APTw, MTRRex AMIDE, MTRRex rNOE, MTRRex ssMT, and the quantitative T1 measurement in the combined GM ROI as well as for the individual regions (FL = frontal lobe, ParTem = parietotemporal lobe, OC = occipital lobe, Ccs = calcarine sulcus). [file NBM-38-e70177-s010.docx]

| GM male | FL | | ParTemp | | OC | | Ccs | | Combined | |
| --- | --- | --- | --- | --- | --- | --- | --- | --- | --- | --- |
|  | mean | SD | mean | SD | mean | SD | mean | SD | mean | SD |
| APTw [%] | 0.23 | 0.16 | 0.59 | 0.34 | 0.28 | 0.36 | 0.99 | 0.65 | 0.45 | 0.30 |
| MTR_Rex_ AMIDE | 0.19 | 0.01 | 0.21 | 0.01 | 0.21 | 0.01 | 0.23 | 0.01 | 0.21 | 0.01 |
| MTR_Rex_ rNOE | 0.30 | 0.01 | 0.32 | 0.02 | 0.32 | 0.01 | 0.32 | 0.02 | 0.31 | 0.01 |
| MTR_Rex_ ssMT | 0.34 | 0.01 | 0.38 | 0.01 | 0.38 | 0.01 | 0.40 | 0.02 | 0.37 | 0.01 |
| T1 [s] | 1.48 | 0.04 | 1.45 | 0.02 | 1.44 | 0.03 | 1.42 | 0.05 | 1.45 | 0.03 |
| GM female | FL | | ParTemp | | OC | | Ccs | | Combined | |
|  | Mean | SD | Mean | SD | Mean | SD | Mean | SD | Mean | SD |
| APTw [%] | 0.09 | 0.48 | 0.29 | 0.67 | 0.01 | 0.77 | 0.98 | 0.79 | 0.21 | 0.66 |
| MTR_Rex_ AMIDE | 0.19 | 0.01 | 0.20 | 0.01 | 0.20 | 0.01 | 0.22 | 0.02 | 0.20 | 0.01 |
| MTR_Rex_ rNOE | 0.28 | 0.01 | 0.31 | 0.01 | 0.31 | 0.01 | 0.31 | 0.01 | 0.30 | 0.01 |
| MTR_Rex_ ssMT | 0.33 | 0.01 | 0.38 | 0.01 | 0.38 | 0.01 | 0.40 | 0.02 | 0.37 | 0.01 |
| T1 [s] | 1.50 | 0.06 | 1.47 | 0.04 | 1.44 | 0.05 | 1.42 | 0.06 | 1.45 | 0.05 |
