# Supplementary material for: CEST Contrasts Exhibit Significant Regional Variations in the Human Brain at 3 T
Source: NMR Biomed. 2025 Nov 13;38(12):e70177. doi: 10.1002/nbm.70177 (PMC12613240; doi:10.1002/nbm.70177)
Supplement: Supplementary file 10 — Table S3: Summary of the median ROI value analysis across all 10 volunteers. Mean and standard deviation (SD) were calculated for APTw,fs, AREX AMIDE, AREX rNOE, AREX ssMT, LD AMIDE, LD rNOE, and LD ssMT and in the combined GM and WM ROIs as well as for the individual regions (FL = frontal lobe, ParTem = parietotemporal lobe, OC = occipital lobe, Ccs = calcarine sulcus). [file NBM-38-e70177-s008.docx]

| GM | FL | | ParTemp | | OC | | Ccs | | Combined | |
| --- | --- | --- | --- | --- | --- | --- | --- | --- | --- | --- |
|  | mean | SD | mean | SD | mean | SD | mean | SD | mean | SD |
| APTw fluidsupp [%] | 0.07 | 0.17 | 0.26 | 0.33 | 0.08 | 0.38 | 0.77 | 0.50 | 0.19 | 0.30 |
| AREX AMIDE [Hz] | 0.13 | 0.01 | 0.14 | 0.01 | 0.14 | 0.01 | 0.16 | 0.01 | 0.14 | 0.01 |
| AREX rNOE [Hz] | 0.20 | 0.01 | 0.22 | 0.01 | 0.22 | 0.01 | 0.22 | 0.01 | 0.21 | 0.01 |
| AREX ssMT [Hz] | 0.23 | 0.01 | 0.26 | 0.01 | 0.26 | 0.01 | 0.28 | 0.02 | 0.26 | 0.01 |
| LD AMIDE | 0.097 | 0.002 | 0.099 | 0.003 | 0.100 | 0.004 | 0.106 | 0.006 | 0.100 | 0.004 |
| LD rNOE | 0.145 | 0.007 | 0.148 | 0.005 | 0.148 | 0.004 | 0.146 | 0.006 | 0.147 | 0.004 |
| LD ssMT | 0.151 | 0.004 | 0.159 | 0.004 | 0.159 | 0.005 | 0.165 | 0.008 | 0.158 | 0.005 |
| WM | FL | | ParTemp | | OC | | Ccs | | Combined | |
|  | Mean | SD | Mean | SD | Mean | SD | Mean | SD | Mean | SD |
| APTw fluidsupp [%] | -0.28 | 0.20 | 0.08 | 0.28 | 0.00 | 0.40 | 0.50 | 0.43 | 0.02 | 0.31 |
| AREX AMIDE [Hz] | 0.15 | 0.01 | 0.16 | 0.01 | 0.16 | 0.01 | 0.17 | 0.01 | 0.16 | 0.01 |
| AREX rNOE [Hz] | 0.27 | 0.03 | 0.26 | 0.01 | 0.26 | 0.01 | 0.26 | 0.02 | 0.26 | 0.01 |
| AREX ssMT [Hz] | 0.36 | 0.04 | 0.35 | 0.01 | 0.36 | 0.02 | 0.36 | 0.03 | 0.36 | 0.02 |
| LD AMIDE | 0.091 | 0.003 | 0.093 | 0.002 | 0.094 | 0.003 | 0.096 | 0.004 | 0.094 | 0.003 |
| LD rNOE | 0.150 | 0.007 | 0.150 | 0.003 | 0.150 | 0.004 | 0.145 | 0.004 | 0.149 | 0.003 |
| LD ssMT | 0.192 | 0.010 | 0.185 | 0.004 | 0.186 | 0.005 | 0.186 | 0.011 | 0.187 | 0.005 |
