# Supplementary material for: CEST Contrasts Exhibit Significant Regional Variations in the Human Brain at 3 T
Source: NMR Biomed. 2025 Nov 13;38(12):e70177. doi: 10.1002/nbm.70177 (PMC12613240; doi:10.1002/nbm.70177)
Supplement: Supplementary file 11 — Table S4: Summary of the median ROI value analysis across all 10 volunteers. Coefficients of variation (CVs) were calculated for MTRRex AMIDE, MTRRex rNOE, MTRRex ssMT, T1, AREX AMIDE, AREX rNOE, AREX ssMT, LD AMIDE, LD rNOE, and LD ssMT and in the combined GM and WM ROIs as well as for the individual regions (FL = frontal lobe, ParTem = parietotemporal lobe, OC = occipital lobe, Ccs = calcarine sulcus). They were not calculated for the APTw contrasts since these metrics are defined on an interval scale. [file NBM-38-e70177-s012.docx]

| GM | FL | ParTemp | OC | Ccs | Combined |
| --- | --- | --- | --- | --- | --- |
|  | CV | CV | CV | CV | CV |
| MTR_Rex_ AMIDE | 0,03 | 0,03 | 0,04 | 0,06 | 0,04 |
| MTR_Rex_ rNOE | 0,05 | 0,05 | 0,04 | 0,05 | 0,04 |
| MTR_Rex_ ssMT | 0,03 | 0,03 | 0,03 | 0,05 | 0,03 |
| T1 [s] | 0,03 | 0,02 | 0,03 | 0,04 | 0,03 |
| AREX AMIDE [Hz] | 0,04 | 0,04 | 0,06 | 0,08 | 0,06 |
| AREX rNOE [Hz] | 0,07 | 0,05 | 0,04 | 0,06 | 0,05 |
| AREX ssMT [Hz] | 0,05 | 0,04 | 0,05 | 0,08 | 0,05 |
| LD AMIDE | 0,02 | 0,03 | 0,04 | 0,05 | 0,04 |
| LD rNOE | 0,05 | 0,04 | 0,03 | 0,04 | 0,03 |
| LD ssMT | 0,03 | 0,03 | 0,03 | 0,05 | 0,03 |
| WM | FL | ParTemp | OC | Ccs | Combined |
|  | CV | CV | CV | CV | CV |
| MTR_Rex_ AMIDE | 0,03 | 0,03 | 0,04 | 0,05 | 0,04 |
| MTR_Rex_ rNOE | 0,05 | 0,03 | 0,03 | 0,04 | 0,03 |
| MTR_Rex_ ssMT | 0,05 | 0,02 | 0,03 | 0,06 | 0,03 |
| T1 [s] | 0,05 | 0,03 | 0,03 | 0,04 | 0,03 |
| AREX AMIDE [Hz] | 0,04 | 0,04 | 0,06 | 0,08 | 0,05 |
| AREX rNOE [Hz] | 0,10 | 0,04 | 0,05 | 0,08 | 0,05 |
| AREX ssMT [Hz] | 0,11 | 0,04 | 0,05 | 0,10 | 0,05 |
| LD AMIDE | 0,03 | 0,02 | 0,03 | 0,04 | 0,03 |
| LD rNOE | 0,04 | 0,02 | 0,03 | 0,03 | 0,02 |
| LD ssMT | 0,05 | 0,02 | 0,03 | 0,06 | 0,03 |
