# Supplementary material for: CEST Contrasts Exhibit Significant Regional Variations in the Human Brain at 3 T
Source: NMR Biomed. 2025 Nov 13;38(12):e70177. doi: 10.1002/nbm.70177 (PMC12613240; doi:10.1002/nbm.70177)
Supplement: Supplementary file 12 — Data S1: Supplementary information. [file NBM-38-e70177-s002.docx]

**Supporting information**

**S1: Representative fits and Z-spectra from all brain regions in GM and WM**


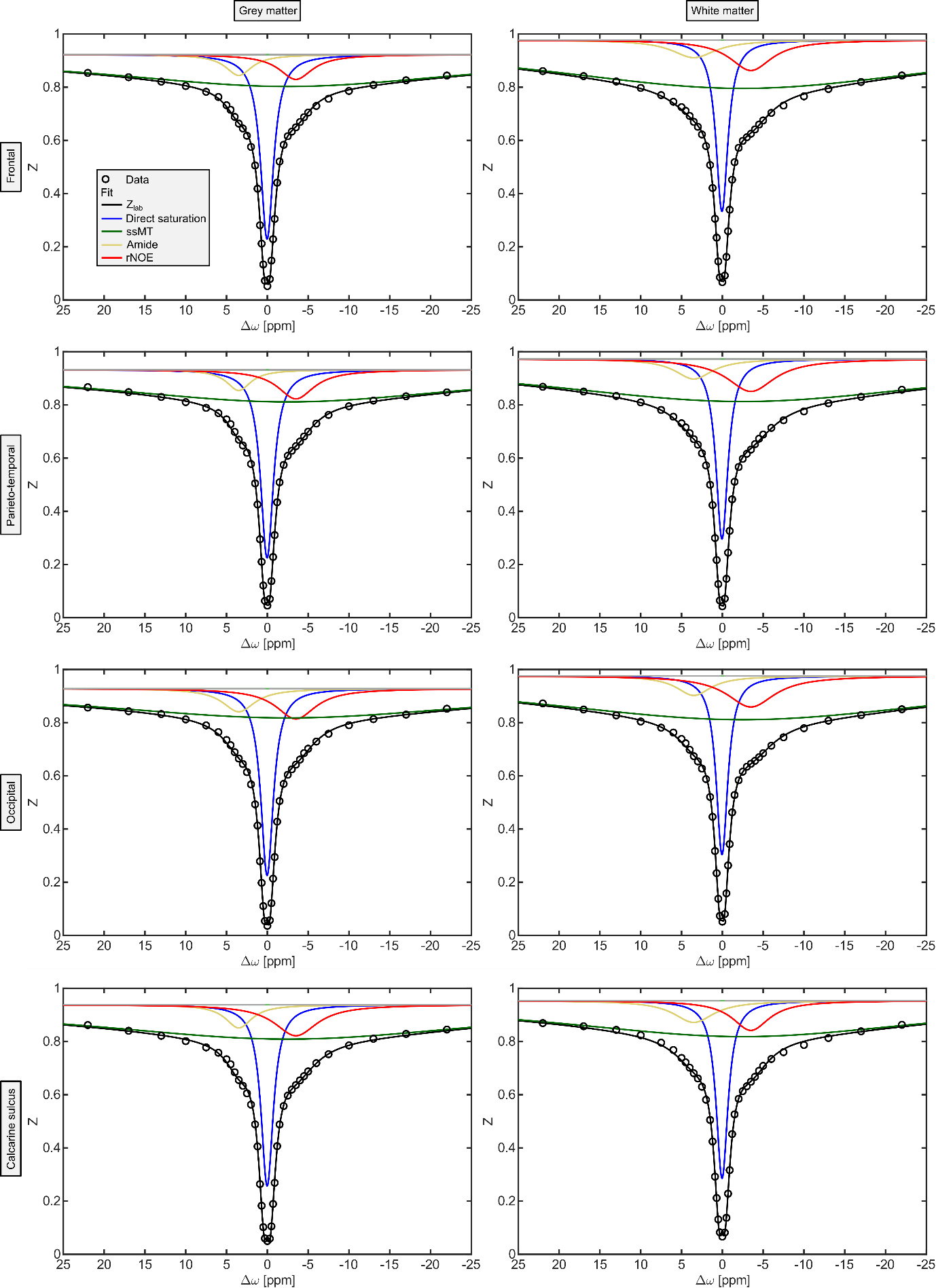


**Figure S1** Z-spectra for gray matter (GM) (left) and white matter (WM) (right) from a healthy volunteer for a representative voxel in each of the evaluated regions (Frontal (top), Parietotemporal (top middle), Occipital (bottom middle), Calcarine sulcus (bottom)). Additionally, the 4-pool Lorentzian-fit (black line) and the direct water saturation (blue), AMIDE (yellow), rNOE (red), and ssMT (green) Lorentz curves are shown. Besides slightly decreased ssMT contributions in the GM ROIs compared to WM, no obvious differences can be observed.

**S2: Boxplot analysis of a second volunteer**

**
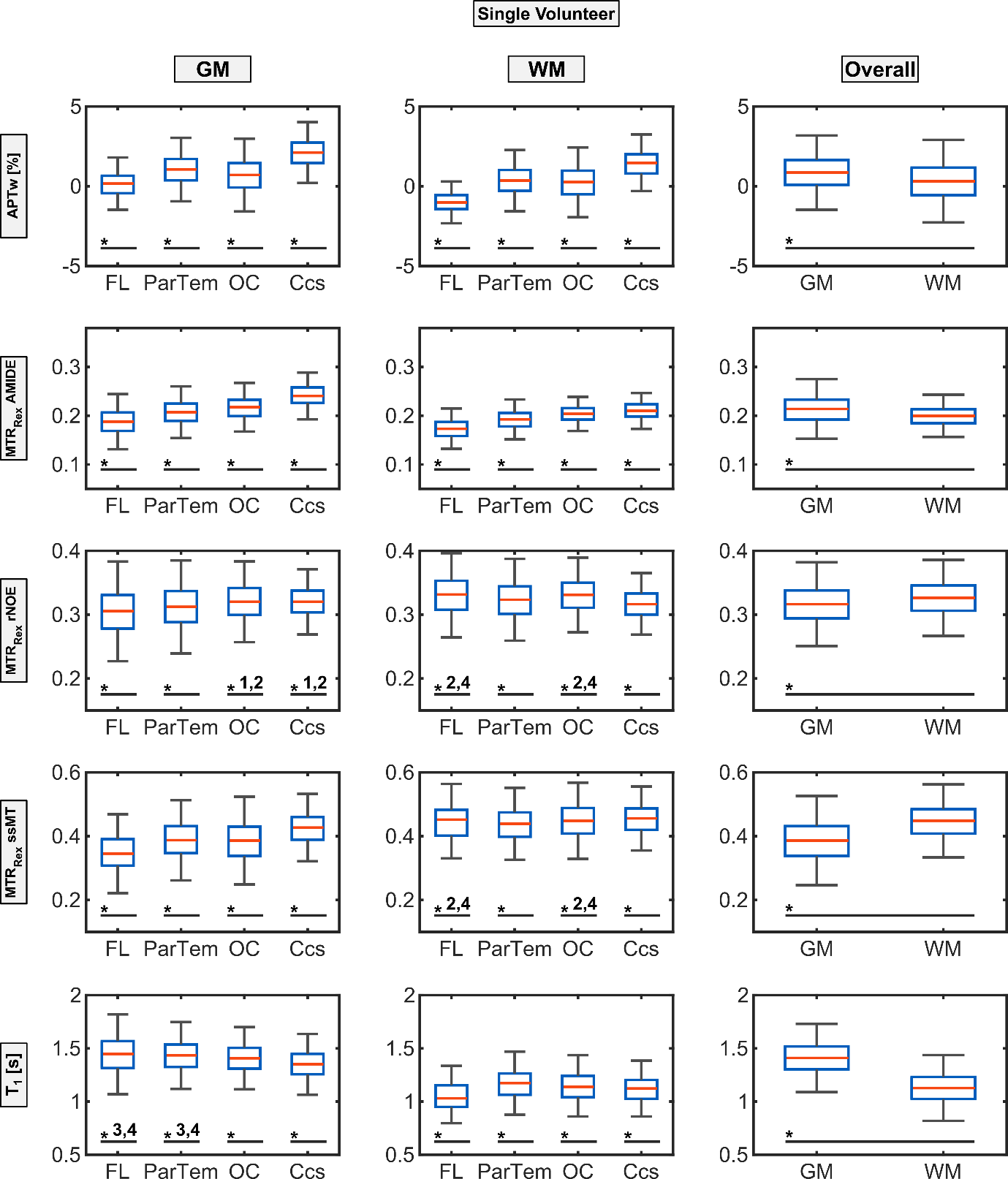
**

**Figure S2** Boxplot showing the ROI analysis of a second representative volunteer as shown in Figure 4. The APTw (first row) and relaxation-compensated CEST contrast values (MTR_Rex_, rows 2-4) as well as T_1_ times (fifth row) are displayed for the GM (left column) and WM (middle column) ROIs located in the frontal lobe (FL, 1) parieto-temporal (ParTem, 2), occipital lobe (OC, 3) and the calcarine sulcus (Ccs, 4). Additionally combined gray matter (GM) ROIs and combined white matter (WM) ROIs (right column) are displayed in the right column. A single asterisk (*) indicates that the marked group differs significantly (p < 0.05) from all other groups within the subfigure. Where an asterisk is followed by a group number (e.g., 2), the marked group differs significantly (p < 0.05) only from that specific group. This analysis matches well with the analysis of the Volunteer shown in Figure 4.

**S3: Analysis of the deviations between male and female volunteers**


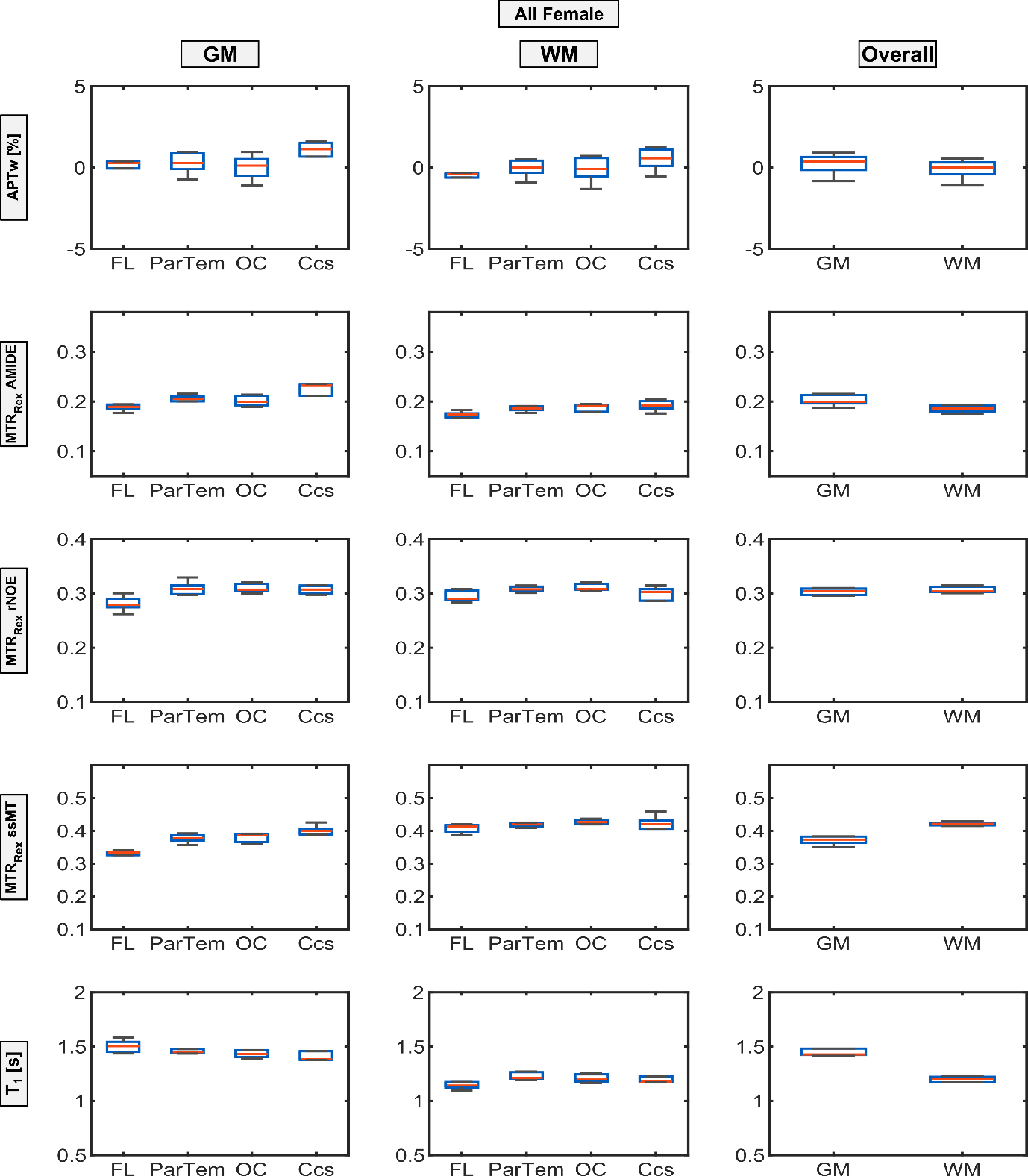


**Figure S3** Boxplot of the median signal values for the APTw (first row) and relaxation-compensated MTR_Rex_ contrasts (rows 2-4) as well as T1 times (fifth row) of all 5 female volunteers (25 ± 4.1 years, Table S1). Data is displayed for the GM (left column) and WM (middle column). ROIs located in the frontal lobe (FL) parieto-temporal (ParTem), occipital lobe (OC) and the calcarine sulcus (Ccs). The combined gray matter (GM) ROIs and combined white matter (WM) ROIs (right column) are displayed in the right column.


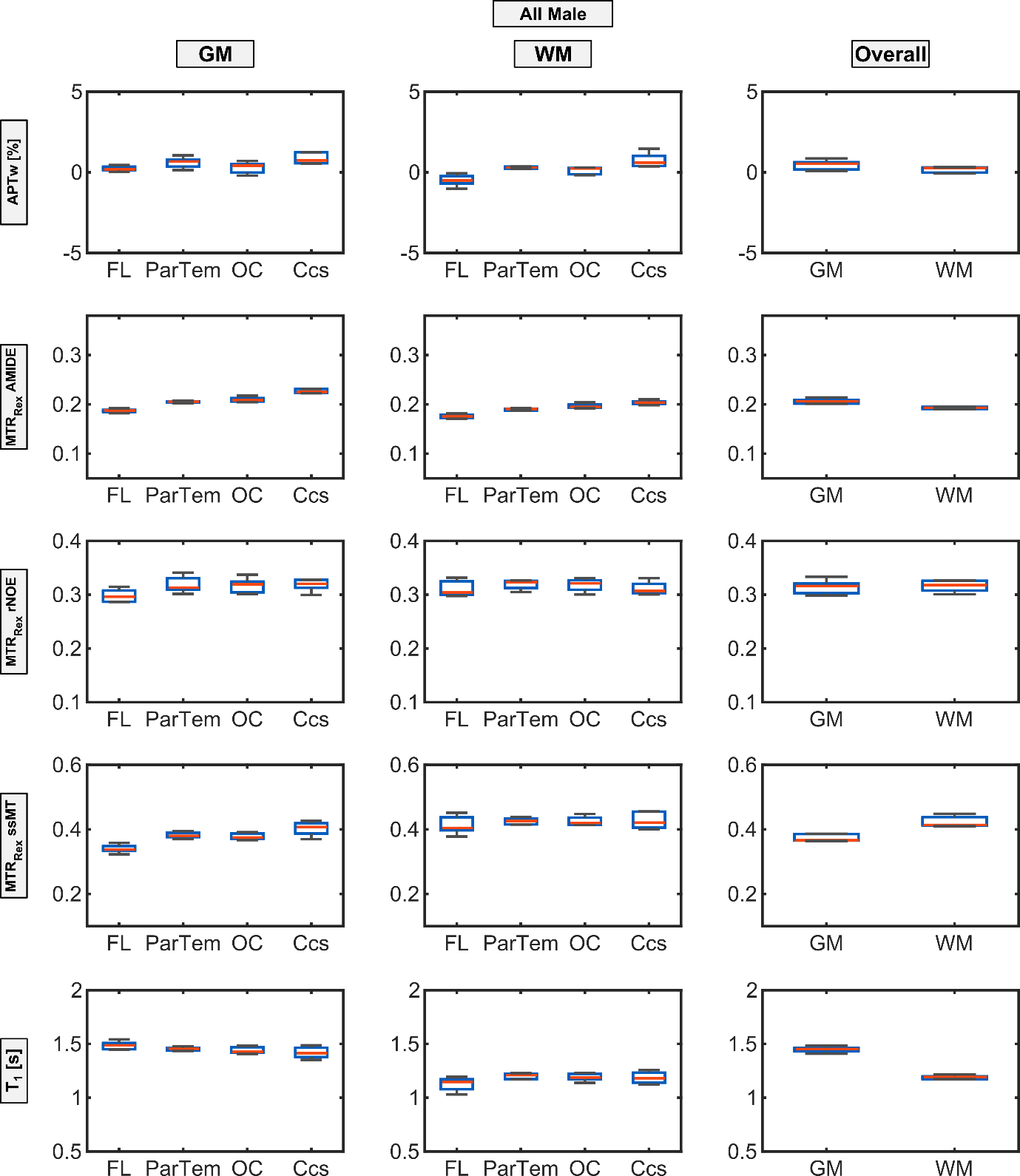


**Figure S4** Boxplot of the median signal values for the APTw (first row) and relaxation-compensated MTR_Rex_ contrasts (rows 2-4) as well as T1 times (fifth row) of all 5 male volunteers (25 ± 1.9 years; Table S2). Data is displayed for the GM (left column) and WM (middle column). ROIs located in the frontal lobe (FL) parieto-temporal (ParTem), occipital lobe (OC) and the calcarine sulcus (Ccs). The combined gray matter (GM) ROIs and combined white matter (WM) ROIs (right column) are displayed in the right column.

**Table S1** Summary of the median ROI value analysis for the GM ROIs across all 5 male (25 ± 1.9 years) and female (25 ± 4.1 years) volunteers. Mean and Standard deviation were calculated for APTw, MTR_Rex_ AMIDE, MTR_Rex_ rNOE, MTR_Rex_ ssMT and the quantitative T1 measurement in the combined GM ROI as well as for the individual regions( FL= frontal lobe, ParTemp= parieto-temporal lobe, OC= occipital lobe, Ccs= calcarine sulcus).

| GM male | FL | | ParTemp | | OC | | Ccs | | Combined | |
| --- | --- | --- | --- | --- | --- | --- | --- | --- | --- | --- |
|  | mean | SD | mean | SD | mean | SD | mean | SD | mean | SD |
| APTw [%] | 0.23 | 0.16 | 0.59 | 0.34 | 0.28 | 0.36 | 0.99 | 0.65 | 0.45 | 0.30 |
| MTR_Rex_ AMIDE | 0.19 | 0.01 | 0.21 | 0.01 | 0.21 | 0.01 | 0.23 | 0.01 | 0.21 | 0.01 |
| MTR_Rex_ rNOE | 0.30 | 0.01 | 0.32 | 0.02 | 0.32 | 0.01 | 0.32 | 0.02 | 0.31 | 0.01 |
| MTR_Rex_ ssMT | 0.34 | 0.01 | 0.38 | 0.01 | 0.38 | 0.01 | 0.40 | 0.02 | 0.37 | 0.01 |
| T1 [s] | 1.48 | 0.04 | 1.45 | 0.02 | 1.44 | 0.03 | 1.42 | 0.05 | 1.45 | 0.03 |
| GM female | FL | | ParTemp | | OC | | Ccs | | Combined | |
|  | Mean | SD | Mean | SD | Mean | SD | Mean | SD | Mean | SD |
| APTw [%] | 0.09 | 0.48 | 0.29 | 0.67 | 0.01 | 0.77 | 0.98 | 0.79 | 0.21 | 0.66 |
| MTR_Rex_ AMIDE | 0.19 | 0.01 | 0.20 | 0.01 | 0.20 | 0.01 | 0.22 | 0.02 | 0.20 | 0.01 |
| MTR_Rex_ rNOE | 0.28 | 0.01 | 0.31 | 0.01 | 0.31 | 0.01 | 0.31 | 0.01 | 0.30 | 0.01 |
| MTR_Rex_ ssMT | 0.33 | 0.01 | 0.38 | 0.01 | 0.38 | 0.01 | 0.40 | 0.02 | 0.37 | 0.01 |
| T1 [s] | 1.50 | 0.06 | 1.47 | 0.04 | 1.44 | 0.05 | 1.42 | 0.06 | 1.45 | 0.05 |

**Table S2** Summary of the median ROI value analysis for the WM ROIs across all 5 male (25 ± 1.9 years) and female (25 ± 4.1 years) volunteers. Mean and Standard deviation were calculated for APTw, MTR_Rex_ AMIDE, MTR_Rex_ rNOE, MTR_Rex_ ssMT and the quantitative T1 measurement in the combined WM ROI as well as for the individual regions( FL= frontal lobe, ParTemp= parieto-temporal lobe, OC= occipital lobe, Ccs= calcarine sulcus).

| WM male | FL | | ParTemp | | OC | | Ccs | | Combined | |
| --- | --- | --- | --- | --- | --- | --- | --- | --- | --- | --- |
|  | mean | SD | mean | SD | mean | SD | mean | SD | mean | SD |
| APTw [%] | -0.49 | 0.36 | 0.29 | 0.07 | 0.11 | 0.22 | 0.74 | 0.45 | 0.16 | 0.18 |
| MTR_Rex_ AMIDE | 0.18 | 0.01 | 0.19 | 0.01 | 0.20 | 0.01 | 0.20 | 0.01 | 0.19 | 0.01 |
| MTR_Rex_ rNOE | 0.31 | 0.01 | 0.32 | 0.01 | 0.32 | 0.01 | 0.31 | 0.01 | 0.32 | 0.01 |
| MTR_Rex_ ssMT | 0.41 | 0.03 | 0.43 | 0.01 | 0.43 | 0.01 | 0.43 | 0.03 | 0.42 | 0.02 |
| T1 [s] | 1.13 | 0.06 | 1.20 | 0.03 | 1.19 | 0.04 | 1.19 | 0.05 | 1.18 | 0.03 |
| WM female | FL | | ParTemp | | OC | | Ccs | | Combined | |
|  | Mean | SD | Mean | SD | Mean | SD | Mean | SD | Mean | SD |
| APTw [%] | -0.54 | 0.39 | -0.02 | 0.56 | -0.09 | 0.80 | 0.54 | 0.71 | -0.10 | 0.61 |
| MTR_Rex_ AMIDE | 0.17 | 0.01 | 0.19 | 0.01 | 0.19 | 0.01 | 0.19 | 0.01 | 0.19 | 0.01 |
| MTR_Rex_ rNOE | 0.29 | 0.01 | 0.31 | 0.01 | 0.31 | 0.01 | 0.30 | 0.01 | 0.31 | 0.01 |
| MTR_Rex_ ssMT | 0.41 | 0.01 | 0.42 | 0.01 | 0.43 | 0.01 | 0.42 | 0.03 | 0.42 | 0.01 |
| T1 [s] | 1.15 | 0.06 | 1.23 | 0.04 | 1.21 | 0.04 | 1.20 | 0.05 | 1.20 | 0.03 |

**S4: Analysis of the regional deviations for the fluid suppressed APTw contrast and the AREX contrasts for AMIDE, rNOE and ssMT**

**
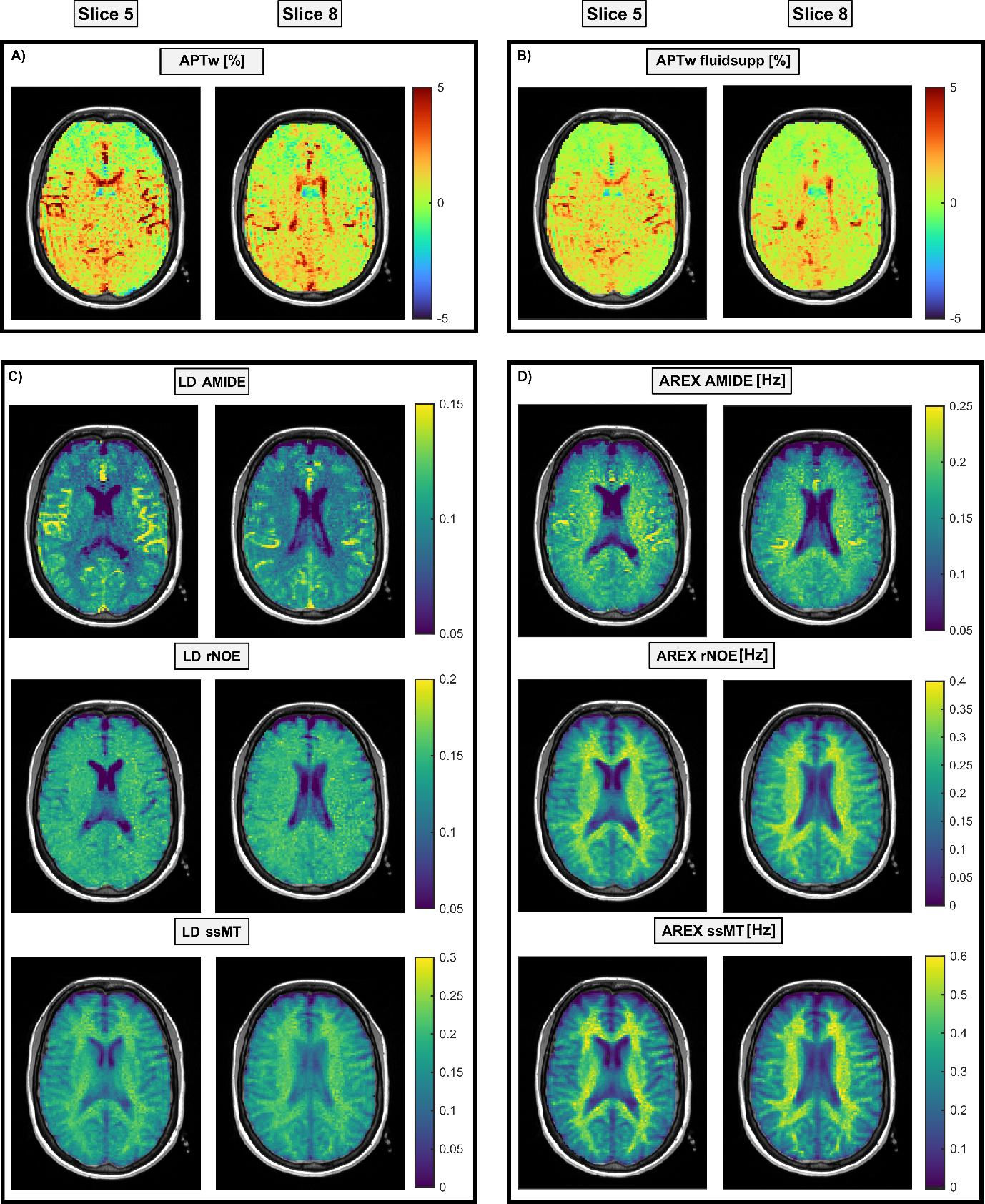
**

**Figure S5** The same two slices as displayed in Figure 1A and 3 are shown for APTw (A), fluid suppressed APTw (B) ,the Lorentzian difference (LD; C) AMIDE, rNOE, and ssMT, and the relaxation-compensated AREX (D) AMIDE, rNOE, and ssMT to present the visual characteristics of the analysed contrasts. The APTw and fluid suppressed APTw show clear differences between GM and WM with an increased GM contrast. Furthermore, Vascular signal are suppressed in the fluid suppressed APTw contrast. In contrast, the AREX rNOE and ssMT show a opposite and T_1_ like behaviour with a clearly increased WM contrast when compared to GM. Lastly, the AREX AMIDE GM-WM contrast behaviour is observably different to the MTR_Rrex_ AMIDE contrasts as it is no longer showing a clear hyperintense GM region and instead a slightly increased contrast in WM. Notably, the fluid suppressed APTw contrast maps depict similar ringing artifacts in the parieto-occipital regions as the APTw contrast. Finally, the LD contrasts show similar characteristics than the MTR_Rex_ contrasts in Figure 3.

**
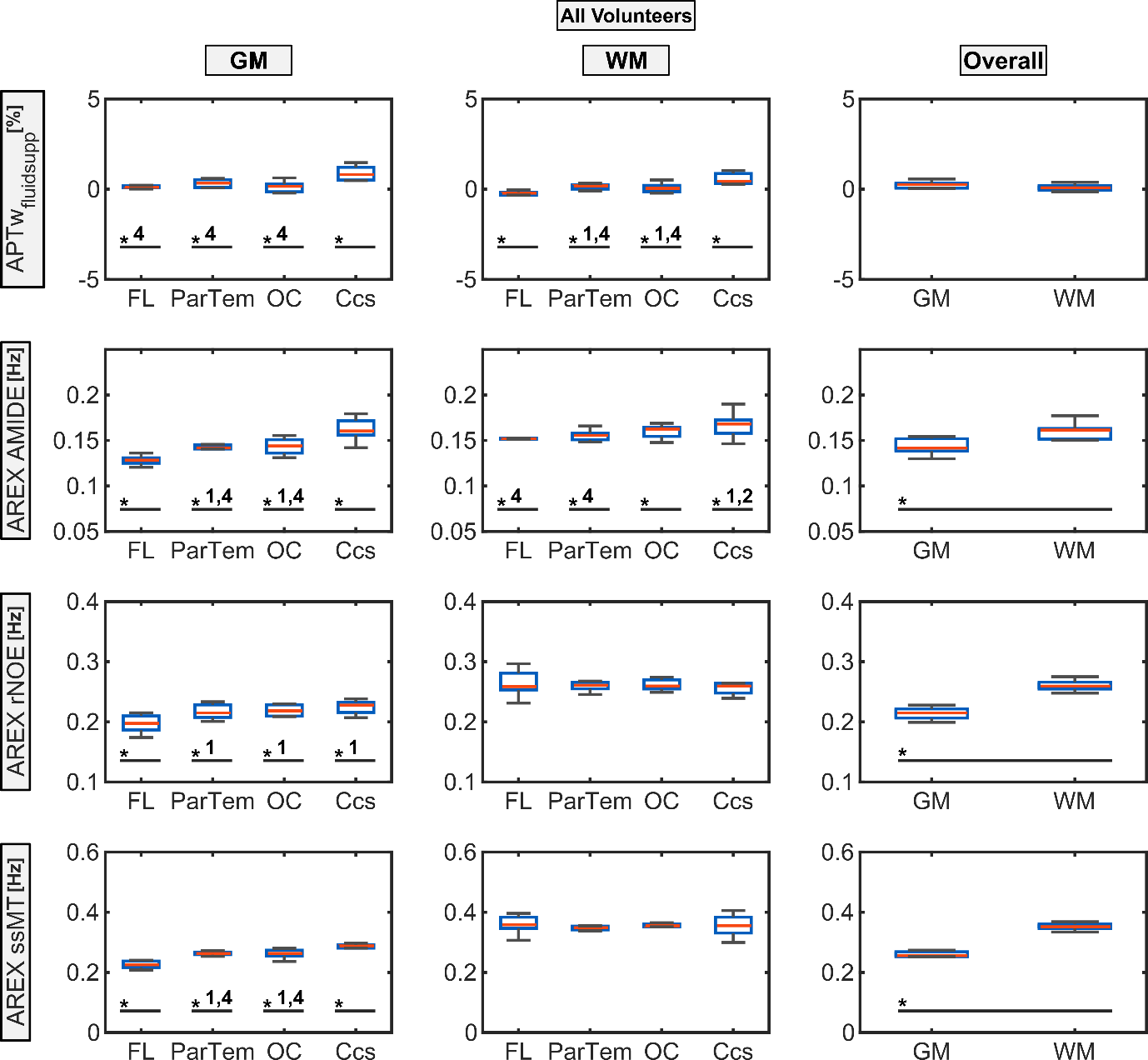
**

**Figure S6:** Boxplot of the median signal values for the APTw _fluidsupp_ (first row) and relaxation-compensated AREX contrasts (rows 2-4) of the 10 volunteers (25 ± 3.1 years, 5 female). Data is displayed for the GM (left column) and WM (middle column). ROIs located in the frontal lobe (FL, 1) parieto-temporal (ParTem, 2), occipital lobe (OC, 3) and the calcarine sulcus (Ccs, 4). The combined gray matter (GM) ROIs and combined white matter (WM) ROIs (right column) are displayed in the right column. A single asterisk (*) indicates that the marked group differs significantly (p < 0.05) from all other groups of ROIs within the subfigure. Where an asterisk is followed by a group number (e.g., 2), the marked group differs significantly (p < 0.05) only from that specific group.

**
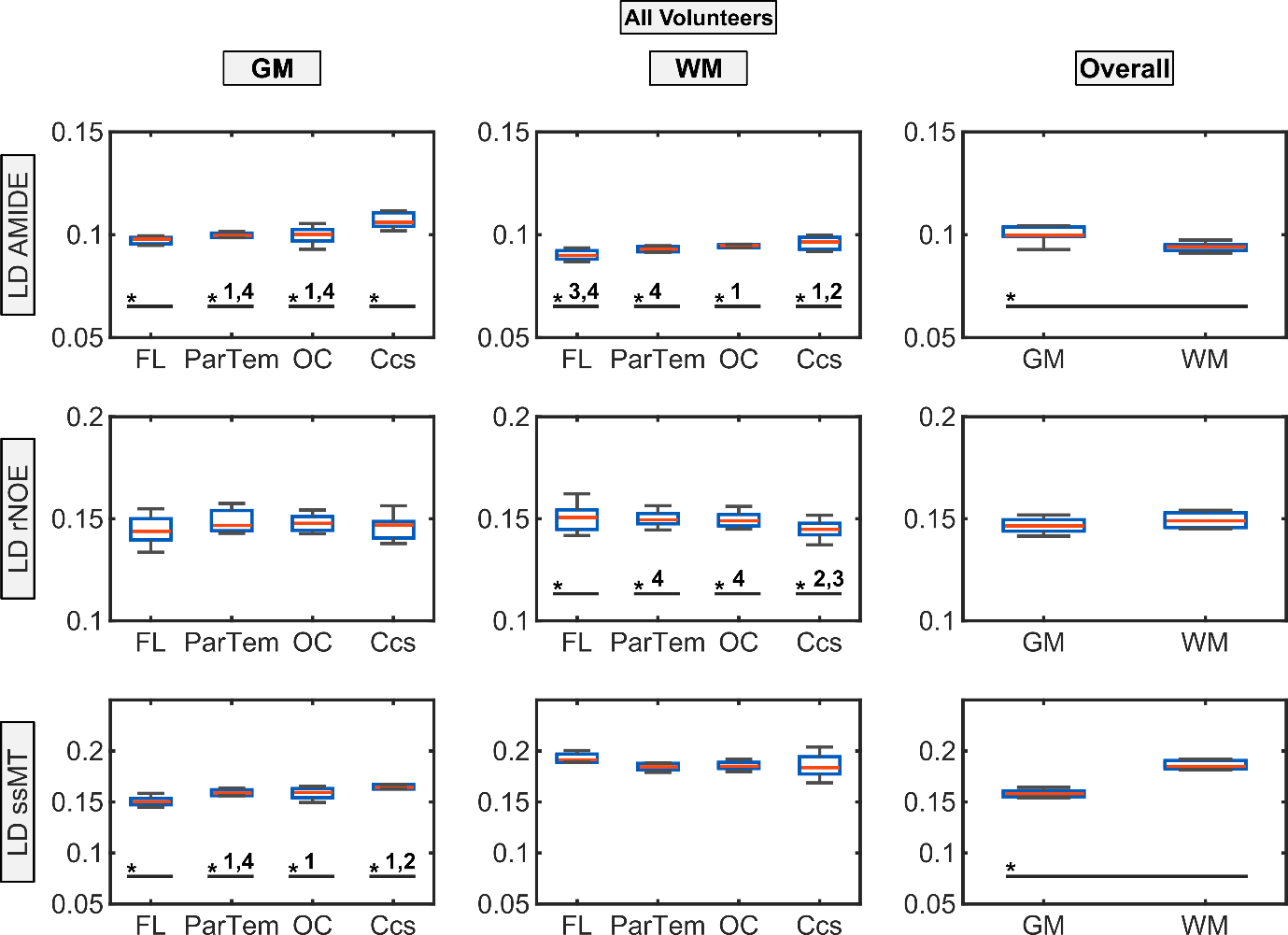
**

**Figure S7:** Boxplot of the median signal values for the LD contrasts (rows 2-4) of the 10 volunteers (25 ± 3.1 years, 5 female). Data is displayed for the GM (left column) and WM (middle column). ROIs located in the frontal lobe (FL, 1) parieto-temporal (ParTem, 2), occipital lobe (OC, 3) and the calcarine sulcus (Ccs, 4). The combined gray matter (GM) ROIs and combined white matter (WM) ROIs (right column) are displayed in the right column. A single asterisk (*) indicates that the marked group differs significantly (p < 0.05) from all other groups of ROIs within the subfigure. Where an asterisk is followed by a group number (e.g., 2), the marked group differs significantly (p < 0.05) only from that specific group.

**Table S3** Summary of the median ROI value analysis across all 10 volunteers. Mean and standard deviation (SD) were calculated for APTw,fs, AREX AMIDE, AREX rNOE, AREX ssMT, LD AMIDE, LD rNOE, and LD ssMT and in the combined GM and WM ROIs as well as for the individual regions (FL= frontal lobe, ParTemp= parieto-temporal lobe, OC= occipital lobe, Ccs= calcarine sulcus).

| GM | FL | | ParTemp | | OC | | Ccs | | Combined | |
| --- | --- | --- | --- | --- | --- | --- | --- | --- | --- | --- |
|  | mean | SD | mean | SD | mean | SD | mean | SD | mean | SD |
| APTw fluidsupp [%] | 0.07 | 0.17 | 0.26 | 0.33 | 0.08 | 0.38 | 0.77 | 0.50 | 0.19 | 0.30 |
| AREX AMIDE [Hz] | 0.13 | 0.01 | 0.14 | 0.01 | 0.14 | 0.01 | 0.16 | 0.01 | 0.14 | 0.01 |
| AREX rNOE [Hz] | 0.20 | 0.01 | 0.22 | 0.01 | 0.22 | 0.01 | 0.22 | 0.01 | 0.21 | 0.01 |
| AREX ssMT [Hz] | 0.23 | 0.01 | 0.26 | 0.01 | 0.26 | 0.01 | 0.28 | 0.02 | 0.26 | 0.01 |
| LD AMIDE | 0.097 | 0.002 | 0.099 | 0.003 | 0.100 | 0.004 | 0.106 | 0.006 | 0.100 | 0.004 |
| LD rNOE | 0.145 | 0.007 | 0.148 | 0.005 | 0.148 | 0.004 | 0.146 | 0.006 | 0.147 | 0.004 |
| LD ssMT | 0.151 | 0.004 | 0.159 | 0.004 | 0.159 | 0.005 | 0.165 | 0.008 | 0.158 | 0.005 |
| WM | FL | | ParTemp | | OC | | Ccs | | Combined | |
|  | Mean | SD | Mean | SD | Mean | SD | Mean | SD | Mean | SD |
| APTw fluidsupp [%] | -0.28 | 0.20 | 0.08 | 0.28 | 0.00 | 0.40 | 0.50 | 0.43 | 0.02 | 0.31 |
| AREX AMIDE [Hz] | 0.15 | 0.01 | 0.16 | 0.01 | 0.16 | 0.01 | 0.17 | 0.01 | 0.16 | 0.01 |
| AREX rNOE [Hz] | 0.27 | 0.03 | 0.26 | 0.01 | 0.26 | 0.01 | 0.26 | 0.02 | 0.26 | 0.01 |
| AREX ssMT [Hz] | 0.36 | 0.04 | 0.35 | 0.01 | 0.36 | 0.02 | 0.36 | 0.03 | 0.36 | 0.02 |
| LD AMIDE | 0.091 | 0.003 | 0.093 | 0.002 | 0.094 | 0.003 | 0.096 | 0.004 | 0.094 | 0.003 |
| LD rNOE | 0.150 | 0.007 | 0.150 | 0.003 | 0.150 | 0.004 | 0.145 | 0.004 | 0.149 | 0.003 |
| LD ssMT | 0.192 | 0.010 | 0.185 | 0.004 | 0.186 | 0.005 | 0.186 | 0.011 | 0.187 | 0.005 |

**Table S4** Summary of the median ROI value analysis across all 10 volunteers. Coefficients of variation (CV) were calculated for MTR_Rex_ AMIDE, MTR_Rex_ rNOE, MTR_Rex_ ssMT, T1, AREX AMIDE, AREX rNOE, AREX ssMT, LD AMIDE, LD rNOE, and LD ssMT and in the combined GM and WM ROIs as well as for the individual regions (FL= frontal lobe, ParTemp= parieto-temporal lobe, OC= occipital lobe, Ccs= calcarine sulcus). They were not calculated for the APTw contrasts since these metrics are defined on an interval scale.

| GM | FL | ParTemp | OC | Ccs | Combined |
| --- | --- | --- | --- | --- | --- |
|  | CV | CV | CV | CV | CV |
| MTR_Rex_ AMIDE | 0,03 | 0,03 | 0,04 | 0,06 | 0,04 |
| MTR_Rex_ rNOE | 0,05 | 0,05 | 0,04 | 0,05 | 0,04 |
| MTR_Rex_ ssMT | 0,03 | 0,03 | 0,03 | 0,05 | 0,03 |
| T1 [s] | 0,03 | 0,02 | 0,03 | 0,04 | 0,03 |
| AREX AMIDE [Hz] | 0,04 | 0,04 | 0,06 | 0,08 | 0,06 |
| AREX rNOE [Hz] | 0,07 | 0,05 | 0,04 | 0,06 | 0,05 |
| AREX ssMT [Hz] | 0,05 | 0,04 | 0,05 | 0,08 | 0,05 |
| LD AMIDE | 0,02 | 0,03 | 0,04 | 0,05 | 0,04 |
| LD rNOE | 0,05 | 0,04 | 0,03 | 0,04 | 0,03 |
| LD ssMT | 0,03 | 0,03 | 0,03 | 0,05 | 0,03 |
| WM | FL | ParTemp | OC | Ccs | Combined |
|  | CV | CV | CV | CV | CV |
| MTR_Rex_ AMIDE | 0,03 | 0,03 | 0,04 | 0,05 | 0,04 |
| MTR_Rex_ rNOE | 0,05 | 0,03 | 0,03 | 0,04 | 0,03 |
| MTR_Rex_ ssMT | 0,05 | 0,02 | 0,03 | 0,06 | 0,03 |
| T1 [s] | 0,05 | 0,03 | 0,03 | 0,04 | 0,03 |
| AREX AMIDE [Hz] | 0,04 | 0,04 | 0,06 | 0,08 | 0,05 |
| AREX rNOE [Hz] | 0,10 | 0,04 | 0,05 | 0,08 | 0,05 |
| AREX ssMT [Hz] | 0,11 | 0,04 | 0,05 | 0,10 | 0,05 |
| LD AMIDE | 0,03 | 0,02 | 0,03 | 0,04 | 0,03 |
| LD rNOE | 0,04 | 0,02 | 0,03 | 0,03 | 0,02 |
| LD ssMT | 0,05 | 0,02 | 0,03 | 0,06 | 0,03 |
